# Functional analysis and identification of miRNAs associated with lipid metabolism from milk-derived exosomes

**DOI:** 10.1186/s40104-025-01331-5

**Published:** 2026-02-04

**Authors:** Xin Lu, Tianyu Deng, Yue Liu, Xiaohan Zhang, Xue Bai, Xibi Fang, Runjun Yang

**Affiliations:** 1 College of Animal Science, Jilin University, No. 5333 Xian Road, Changchun, 130062 People’s Republic of China; 2 College of Animal Science and Veterinary Medicine, Tianjin Agricultural University, Tianjin, 300384 People’s Republic of China

**Keywords:** Dairy cattle, Lipid metabolism, Milk-derived exosomes, Milk fat percentage, miRNA

## Abstract

**Background:**

Exosomes are crucial mediators of intercellular communication. As a key component of milk, milk-derived exosomes are abundant in genetic cargo, particularly microRNAs (miRNAs), indicating their potential role in regulating mammary gland physiology. Therefore, this study aimed to investigate the specificity of miRNAs in milk-derived exosomes and their regulatory roles in lipid synthesis in bovine mammary epithelial cells (BMECs).

**Results:**

Based on 17,838 DHI records showing a significantly higher milk fat percentage (MFP) in late lactation (4.24% ± 1.07%), 10 high- (5.96% ± 0.26%, HMF) and 10 low-MFP (1.68% ± 0.23%, LMF) cows were selected during this stage for milk-derived exosome isolation and miRNA profiling. Exosomes isolated via differential ultracentrifugation were verified as 50–150 nm vesicles expressing CD9, CD81, and TSG101. miRNA sequencing identified 1,320 differentially expressed miRNAs (496 upregulated and 824 downregulated) between the HMF_EXO and LMF_EXO groups. Uptake assays confirmed that BMECs internalized these exosomes, and qRT-PCR validation showed that miR-423-5p and miR-125b were significantly upregulated and downregulated in HMF_EXO- and LMF_EXO-treated BMECs, respectively. Functionally, exosomal miR-423-5p promoted intracellular lipid accumulation and TG synthesis in BMECs by targeting *APOA5*, whereas miR-125b inhibited lipolysis and fatty acid oxidation by repressing *SLC27A1*.

**Conclusions:**

This study demonstrates that milk-derived exosomal miRNAs represent a novel mechanism for regulating milk fat synthesis. Specifically, miR-423-5p and miR-125b directly modulated lipid metabolism in BMECs via the miR-423-5p/*APOA5* and miR-125b/*SLC27A1* pathways. These findings provide new insights into the molecular regulation of milk fat synthesis and highlight the importance of exosome-mediated intercellular communication in the lactating mammary gland.

**Graphical Abstract:**

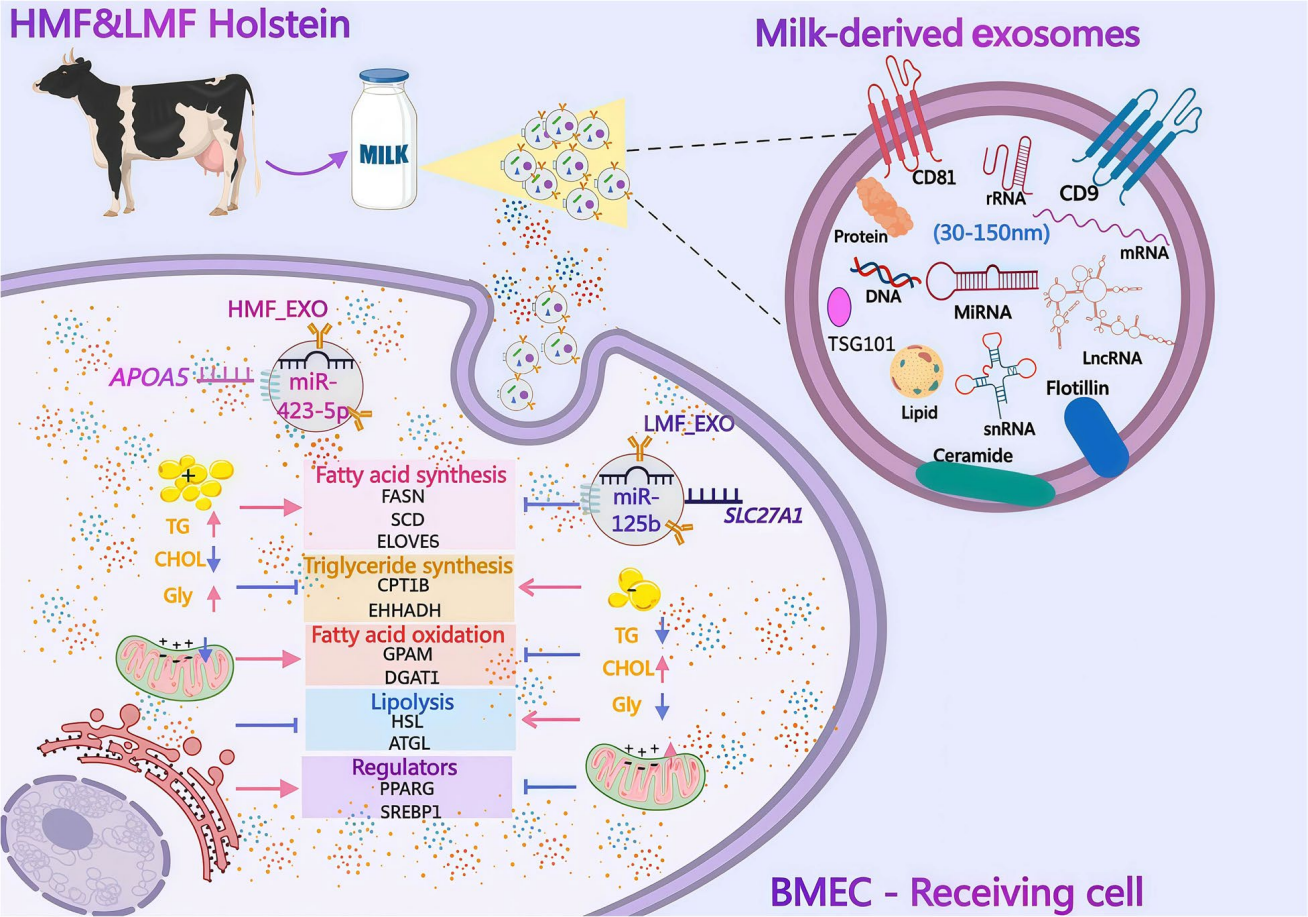

**Supplementary Information:**

The online version contains supplementary material available at 10.1186/s40104-025-01331-5.

## Introduction

Milk fat percentage (MFP) is a key indicator of milk quality assessment [1]. As one of the leading nutritional components of milk, milk fat provides people with high-quality natural fat [2]. Milk with a high fat content has a better taste and richer flavor, and it serves as an essential source of raw materials for processed foods, such as cream, condensed milk, cheese, and butter [3, 4]. The MFP trait is highly heritable [5, 6], and milk fat synthesis is a dynamic and complex multi-network regulatory process that requires the involvement of a large number of genes [7, 8]. The milk fat content of common dairy breeds, such as Holstein cows, typically ranges from 3.6% to 4.0%, whereas that of Guernsey cows ranges from 4.5% to 6.0% [9]. Numerous key components are involved in milk fat metabolism and synthesis [10], including genes such as *FASN*, *ACACA*, and *SCD* [11], which are responsible for de novo fatty acid synthesis. *LPL* and *VLDLR* are involved in fatty acid uptake, transport, and activation [12]. Previous genetic analyses have identified genes associated with milk components. Transcriptome profiling and differential expression gene analyses have been conducted using RNA-seq in mammary tissues of high- and low-milk-producing cows at different lactational stages, as well as between lactation and non-lactation cows. The identified candidate genes for milk production traits include *LPL*, *SCD*,* GPAM*, *LPIN1*, *DGAT*, and *AGPAT6* [13, 14]. Furthermore, signaling pathways and transcription factors, such as mTOR, *SREBP*, *PPARG*, and *AMPK*, are integral to the regulation of milk fat metabolism and synthesis [15, 16]. In milk fat metabolism, miRNAs have been reported to affect the lipid content in bovine mammary epithelial cells (BMECs) by regulating fatty acid synthesis, transport, and oxidation [17]. Studies have demonstrated that miRNAs regulate lipid metabolism by targeting specific genes. For example, miR-29b targets *LPL* and *TDG* [18], miR-2382-5p targets *NDRG2* to promote lipid synthesis in BMECs [19], and miR-423-5p targets *FAM3A* to affect hepatic glycolipid metabolism [20]. MiR-125b targets *ACC* and *FASN* to promote hepatic lipid accumulation via the fatty acid synthesis pathway [21].

Exosomes, nanoscale extracellular vesicles (30–150 nm), are now recognized as key players in intercellular communication [22]. Exosomes originate from the fusion of multivesicular bodies with the plasma membrane [23], which are secreted by various cells, including stem cells, immune cells, and cancer cells [24]. Recently, exosomes have emerged as novel regulators of intercellular communication. Exosomes are enriched with a diverse array of proteins, lipids, and nucleic acids [25]. The mRNA, miRNA, circRNA, and lncRNA within exosomes are remarkably diverse and play critical roles in various biological processes [26]. Once considered mere cellular debris, EVs have been reclassified as vital molecular messengers, shuttling proteins, metabolites, and nucleic acids throughout the body [27]. Recent studies have suggested that exosomes are involved in intercellular communication [28], immune response [29], tissue repair and regeneration [30], and lipid metabolism [31]. Exosomes can also serve as biological carriers that directly transport lipids, including fatty acids, eicosanoids, and CHOL [32]. Milk is a rich source of these vesicles, which carry a diverse payload of genetic material, including proteins, lipids, and nucleic acids [33]. They play important roles in nutrient delivery and immune regulation [34]. Milk-derived exosomal miRNAs facilitate intercellular communication and modulate the gut microbiota, thereby playing regulatory roles in key biological processes, such as lipid metabolism [35] and immune responses [36, 37]. Buffalo milk exosomes alleviate high-fat diet-induced liver lipid disorders in mice by reducing lipid deposition and improving their serum lipid profiles [38]. Recent studies have demonstrated that milk-derived exosomal miRNAs play a significant role in regulating systemic metabolism, including the modulation of glucose metabolism and lipid homeostasis [39]. Of particular interest is whether these exosomes exert paracrine regulatory effects on mammary epithelial cells; however, their functional involvement in milk fat synthesis remains poorly understood and warrants further investigation. Milk-derived exosomes carry miR-148a and other bioactive components that actively participate in the regulation of systemic lipid metabolism by promoting adipocyte proliferation and differentiation [12]. Exosomal miR-11987 promotes the browning of white adipocytes and enhances mitochondrial energy metabolism, thereby reducing body weight gain and offering a potential therapeutic strategy for obesity [40]. These exosomal molecules also contribute to epigenetic regulation and neuroimmune modulation, highlighting their multifaceted biological roles.

Despite growing evidence that exosomal miRNAs regulate lipid metabolism, it remains unclear whether milk-derived exosomes from cows with different milk fat contents differ in their miRNA composition and functions. In this study, we profiled and functionally characterized miRNAs in milk-derived exosomes from cows with high and low milk fat content using an in vitro BMEC model, providing mechanistic insights into their roles in milk fat synthesis.

## Materials and methods

### Collection of DHI data

This study focused on milk samples and DHI data of Holstein dairy cows from a farm in Fuyu County, Heilongjiang Province, China. A total of 17,838 DHI records from January 2021 to February 2023 were collected, encompassing data on milk yield, milk fat percentage, 4% fat-corrected milk yield (4% FCM), milk protein percentage (MPP), somatic cell count (SCC), somatic cell score (SCS), and milk urea nitrogen (MUN) levels. The stages of lactation were categorized as follows: early lactation (0–99 d, *n* = 2,035), mid-lactation (100–199 d, *n* = 2,894), late lactation (200–299 d, *n* = 1,783), and terminal lactation (≥ 300 d, *n* = 924). For each of the four stages, comparative analysis, ANOVA, and correlation analysis were performed. Based on these analyses, dairy cows in late lactation and those with two or more calvings were identified as candidate subjects. Milk fat percentages higher than 4.5% for the high milk fat group and lower than 3% for the low milk fat group were chosen for sampling.

### Ultracentrifugal differential centrifugation of milk-derived exosomes

**Fig. 1:**
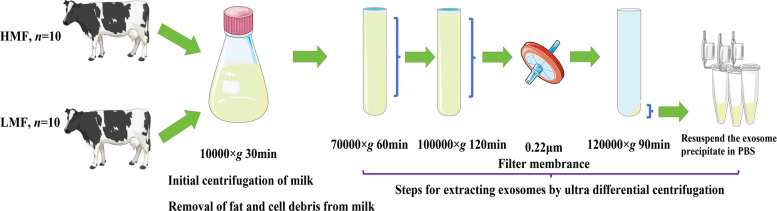
Workflow of ultracentrifugal differential centrifugation of milk-derived exosomes. Image provided by Servier Medical Art (https://smart.servier.com/), licensed under CC BY 4.0 (https://creativecommons.org/licenses/by/4.0/) and BioGDP.com (https://biogdp.com/)

The workflow for obtaining milk-derived exosomes is shown in Fig. 1. Briefly, milk collected from the dairy farm was centrifuged at 300 × *g* for 10 min, 3,000 × *g* for 20 min, and 10,000 × *g* for 30 min at 4 °C to remove milk fat and mammary epithelial cells. Milk samples were stored at −80 °C freezer and thawed at 4 °C. The milk samples were centrifuged at 30,000 × *g* for 60 min at 4 °C to collect the supernatant and discard the precipitate (Beckman Coulter Optima XPN-100 ultracentrifuge, 70Ti). The supernatant was centrifuged at 50,000 × *g* for 60 min at 4 °C to collect two-thirds of the supernatant and discard the precipitate. The collected supernatant was centrifuged at 70,000 × *g* for 60 min at 4 °C. Two-thirds of the supernatant was collected and the precipitate was discarded. The supernatant was filtered through a 0.45-μm filter and centrifuged at 95,000 × *g* for 60 min at 4 °C. Two-thirds of the supernatant was collected, filtered through a 0.22-μm filter (Millipore, Amicon Ultura 100 kDa, USA), and centrifuged at 120,000 × *g* for 90 min at 4 °C to collect the precipitate. After resuspending the milk-derived exosomes in 200 μL of PBS, they were transferred to a clean PE tube and stored at −80 °C.

### Nanoparticle tracking analysis and electron microscopy experiments

Exosomes (10 µL) were pipetted and thoroughly mixed with 1 mL PBS. The diluted exosome resuspension was tested on a Zetasizer Nano (Malvern Panalytical, England) at room temperature, and the particle size range of the exosomes was recorded. The exosome suspension (20 μL) was added to a carbon-coated copper grid (ZXBR, Beijing, China). After 3–5 min, the excess liquid was removed. Next, 2% phosphotungstic acid was added for 1–2 min of staining. Excess stain was removed, and the grid was air-dried at room temperature. Finally, the grid was examined using transmission electron microscopy (TEM, Hitachi, Tokyo, Japan).

### MiRNA-seq analysis and verification of milk-derived exosomes

Exosomal RNA was extracted from milk-derived exosomes using the Norgen Exosomal RNA Isolation Kit (Norgen Biotek Corp., Thorold, Canada). After the extracted exosomal RNA passed the quality check, it was used for subsequent miRNA-seq and library construction. The miRNA-seq process was performed according to the standard steps provided by Illumina, including library preparation and sequencing experiments. The sRNA sequencing library was prepared using the TruSeq Small RNA Sample Prep Kit (Illumina, San Diego, USA) by LC-Bio Technology Co., Ltd. (Hangzhou, China). After library preparation work is completed, the constructed library was sequenced using Illumina HiSeq 2000/2500, and the sequencing read length was single-end 50 bp (SE50). ACGT101-miR (v4.2, LC-Bio Technology, Hangzhou, China) was used for miRNA data analysis. The analysis process was as follows: (1) removal of the 3′ adapter and junk sequences to obtain Clean Data; (2) sRNA length screening to retain the length range of 18–26 nt; (3) comparison and analysis of various RNA databases: alignment of the remaining sequences to mRNA, RFam, and Repbase databases (excluding miRNA) and filtering; (4) miRNA identification: obtaining reads after length filtering and filtering by databases such as RFam, and performing miRNA identification by aligning precursors and genomes; (5) miRNA differential expression analysis; and (6) target gene prediction and target gene enrichment analysis of differentially expressed miRNAs (DEmiRNAs). In this experiment, the miRNA-seq data were analyzed for differential expression. miRNAs with |log_2_FC| > 1, *P-*value < 0.05, and norm > 100 were identified as differentially expressed regulators.

### Uptake of exosomes in BMECs

The diluent from the PKH67 kit (BBcellProbe, Bestbio, Shanghai, China) was used to dilute the probe 10-fold. Opti-MEM was used to dilute the solution 25-fold to prepare the staining working solution. The milk-derived exosomes were resuspended in staining working solution and incubated at 37 °C in the dark for 30 min. After incubation, the suspension was diluted to 40 mL with PBS and subjected to ultracentrifugation to obtain stained exosomes. The pellet was resuspended in 200 µL of PBS.

BMECs were seeded in 24-well plates in advance, and round cell coverslips were placed into the wells. Stained exosomes were added when the cell density reached approximately 80%. After approximately 12 h, when cells reached nearly 100% confluence, they were washed with cold PBS. Subsequently, cells were fixed with 4% paraformaldehyde on ice for 15 min. The cells were then permeabilized with PBS containing 0.5% Triton X-100 at room temperature for 10 min. One microliter of Phalloidin (Solarbio, Beijing, China) stock solution was diluted with 200 μL of PBS and incubated in each well of cells at room temperature for 20 min. The cells were then stained with DAPI (Beyotime, Shanghai, China) for 1 min. Finally, the coverslips were mounted onto glass slides using an anti-fade fluorescence mounting medium (P0126, Beyotime, Shanghai, China) for subsequent observation under fluorescence microscopy (Nikon TE2000, Tokyo, Japan) and Laser Scanning Confocal Microscopy (ZEISS ZEN 3.8., Germany).

### Construction of vectors

The mimics of miR-423-5p, miR-125b, mimic NC, inhibitor of miR-423-5p and miR-125b, and inhibitor negative controls (inhibitor-NC) were chemically synthesized by Integrated Biotech (IBSBIO, Shanghai, China) based on the mature sequences of bta-miR-423-5p and bta-miR-125b retrieved from the miRBase database (http://www.mirbase.org/, Table S1). Following synthesis, 1 OD of lyophilized oligo RNA was centrifuged at 12,000 × *g* for 5 min at 4 °C, resuspended in 125/250 μL of DEPC water, vortexed thoroughly, aliquoted into light-protected tubes, and stored at −80 °C for subsequent experiments.

Based on the *Bos taurus* mRNA sequences of *APOA5* (NM_001077909.1) and *SLC27A1* (NM_001077909.1) from the NCBI database (https://www.ncbi.nlm.nih.gov/), specific restriction sites (MluI and NotI for *APOA5*, BamHI and NotI for *SLC27A1*) were introduced at both ends of the CDS region. DNA fragments were synthesized by Genewiz (Suzhou, China) and subsequently cloned into the corresponding digested (MluI/NotI or BamHI/NotI) pBI-CMV3 vector backbone, resulting in the construction of the pBI-CMV3-*APOA5* and pBI-CMV3-*SLC27A1* over-expression vectors.

The shRNA sequences targeting *SLC27A1* and *APOA5* were designed using the ThermoFisher RNAi Designer tool (https://rnaidesigner.thermofisher.com/rnaiexpress; Waltham, MA, USA). EcoRI and BamHI restriction sites were incorporated at the 5′ termini of the oligonucleotides, which were synthesized by Sangon Biotech (Shanghai, China; primer details are listed in Table S2). Synthesized DNA oligos were annealed to generate double-stranded DNA fragments. These fragments were ligated into a linearized pshRNA-copGFP lentivector (System Biosciences, CA, USA) using T4 DNA ligase (Takara, Dalian, China).

### Cell culture and transfection

The BMECs used in this experiment were derived from cells previously isolated and preserved by our research group [41]. The experimental procedure for miRNA transfection into cells was performed according to the Lipofectamine 3000 transfection protocol (Invitrogen, Carlsbad, CA, USA).

### RNA extraction and quantitative real-time PCR

Total RNA was extracted from milk-derived exosomes using the RNAiso Plus reagent (Takara, Dalian, China) and RNA-EZ Reagents K RNA-Be-Down (Sangon Biotech, Shanghai, China). cDNA was synthesized using a reverse transcription kit (Tolobio, Shanghai, China), with 2 µg of total RNA, 4 µL of 5 × all-in-one RT Buffer, 1 µL of all-in-one enzyme mix, and RNase-free ddH_2_O added to make a total volume of 20 µL. The cDNA synthesis conditions were as follows: incubation at 50 °C for 15 min and 85 °C for 5 s. The specialized kit for the first-strand cDNA synthesis of miRNA is the miRNA 1st Strand cDNA Synthesis Kit (by Tailing A, Tolobio, Shanghai, China). Under the action of Poly A Polymerase (PAP), the tailing reverse transcription reaction was performed after adding poly (A) to the 3′-end of the template. According to the manufacturer's instructions, the miRNA reverse transcription system consists of the following components: 2 µg of total RNA, 5 µL of 2 × miR RT buffer (tailing), 1 µL of ToloScript III miR enzyme mix (tailing) and RNase-free ddH_2_O were added to a total volume of 10 µL of the reaction mixture. cDNA synthesis was carried out by incubation at 37 °C for 60 min, followed by heating at 85 °C for 5 min.

qRT-PCR was performed using SYBR qPCR Master Mix (Tolobio, Shanghai, China) with specific primers listed in Table S1. qRT-PCR was performed in a 10-µL reaction with 0.25 µL of forward and reverse primers, 5 µL of 2 × Q3 SYBR qPCR Master Mix (Universal) (Tolobio, Shanghai, China), 1 µL of cDNA, and 3.5 µL of ddH_2_O. The following procedure was used: 95 °C for 3 min, 40 cycles of 95 °C for 10 s, and 60 °C for 30 s in PCRmax (Eco, Staffordshire, UK). Technical and biological replicates were performed three times each, and *β-actin* was used as an internal standard to normalize mRNA expression levels using the 2^−∆∆CT^ method.

### Detection of intracellular lipid constituents

Triglyceride (TG), total cholesterol (CHOL), and glycerin levels were quantified using specialized detection kits (Tissue/Cell Triglyceride Content Enzymatic Determination Kit E1013 and Total Cholesterol Assay Kit E1015, Applygen Technologies Inc., Beijing, China) according to the manufacturer's instructions. Post-transfection, the cells were processed through enzymatic digestion, centrifugation, and collection before being treated with lysis buffer (incubated on ice for 10 min). A total of 10 µL aliquots were reserved for protein concentration normalization using the Enhanced BCA Protein Quantitation Assay Kit (KGP902, KeyGEN BioTECH, Nanjing China). The residual lysate was subjected to thermal processing (70 °C, 10 min), followed by centrifugation (2,000 r/min, 5 min, room temperature). The supernatants were analyzed photometrically using a multifunctional microplate reader (Biotech, USA), with triplicate measurements across three independent experimental replicates. The final TG concentrations were normalized against the corresponding protein levels (mg protein basis). Post-transfection (48 h), the cells were harvested and processed using the Free Aliphatic Acid Content Assay Kit (Solarbio, Beijing, China). Cells were washed with ice-cold PBS, trypsinized (0.25%), and lysed in 500 μL of extraction buffer. Following the manufacturer's instructions, the supernatant was collected after centrifugation (8,000 r/min, 10 min, 4 °C). Aliquots of 30 μL of lysate were mixed with 300 μL Reagent 1 and 120 μL Reagent 2 (pre-warmed at 40 °C), vortexed (15 min), and centrifuged (3,000 r/min, 10 min). The upper phase (50 μL) was reacted with 200 μL Reagent 3 for 5 min, incubated (15 min), and absorbance was measured at 550 nm using a multimode microplate reader (Tecan Spark, Männedorf, Switzerland).

### Oil Red O and BODIPY staining

Oil Red O staining is currently the most common method for estimating lipid accumulation. Briefly, the cells were gently rinsed twice with cold PBS and fixed in paraformaldehyde (Biosharp, Hefei, China) at 37 °C for 30 min. The cells were then washed with PBS and stained with Oil Red O (Sigma, Shanghai, China) at room temperature for 20 min. After staining, the cells were soaked in 60% isopropanol (Beijing Chemical Works, Beijing, China) for 30 s and stained in the dark for 5 min with hematoxylin. The cells were then observed under a microscope (Olympus, Tokyo, Japan) within 1 h.

The cells were cultured on coverslips pre-placed in 12-well plates. After 2 d of transfection and 2 d of induction, BODIPY staining was performed as follows: the culture medium was removed and replaced with BODIPY (Thermo Fisher Scientific, Waltham, USA) working solution (prepared fresh by mixing 1,999 μL growth medium with 1 μL BODIPY stock solution; stock solution: 1 mg BODIPY dissolved in 250 μL DMSO, aliquoted in light-protected tubes, and stored at −20 °C). After 30 min incubation in darkness, the staining solution was discarded. The cells were gently washed three times with ice-cold PBS and fixed with 4% paraformaldehyde for 5 min. After removing the fixative and repeating PBS washes, the nuclei were counterstained with DAPI for 10 min. After DAPI removal and final PBS rinses, the samples were imaged using a fluorescence microscope (TE2000; Nikon, Tokyo, Japan). All washing procedures employed ice-cold PBS with gentle handling to preserve the cellular integrity.

### EdU staining and Cell Counting Kit-8 (CCK8)

BMECs were seeded in 24-well and 96-well cell culture plates and transfected when the cell density increased to 60%–70%. Cell proliferation was detected after 48 h. EdU staining was performed using the BeyoClick™ EdU Cell Proliferation Kit with Alexa Fluor 488/555 (Beyotime, Shanghai, China). Fluorescence microscopy (Nikon, Tokyo, Japan) was used for imaging. The CCK8 assay was performed using the CellTiter96 AQueous One Solution (Promega, Madison, WI, USA). After adding 20 μL of the reagent to 100 μL of the culture medium in each well of the 96-well plate, the plates were incubated for 2 h at 37 °C in a 5% CO_2_ atmosphere. The absorbance values were measured at 450 nm using an Eon Microplate Spectrophotometer (BioTek, Winooski, VT, USA).

### Membrane potential monitoring

The BMECs were seeded in 24-well plates. Mitochondrial membrane potential was assessed 48 h post-transfection using the JC-1 mitochondrial membrane potential assay kit (HY-K0601, MedChemExpress) according to the manufacturer's instructions. The pre-aliquoted JC-1 solution (200 μmol/L) was equilibrated to room temperature before use. Subsequently, 5 μL JC-1 (200 μmol/L) was added to each well to achieve a final concentration of 2 μmol/L. The plates were gently mixed and incubated at 37 °C for 20 min. The cells were washed with cold PBS and immediately observed under a fluorescence microscope. The excitation wavelengths were set at 488 nm (green monomer) and 585 nm (red polymer), corresponding to emission wavelengths of 530 nm and 590 nm, respectively. Changes in mitochondrial membrane potential were quantified by calculating the ratio of red to green fluorescence intensity (FL2/FL1). Normal cells predominantly exhibited red fluorescence, whereas apoptotic cells exhibited enhanced green fluorescence. A decreased FL2/FL1 ratio indicates a reduction in mitochondrial membrane potential. To ensure accuracy, light exposure was minimized throughout the procedure and detection was completed within 30 min to prevent fluorescence quenching. Finally, the data were analyzed using ImageJ to determine the proportion of cells with reduced mitochondrial membrane potential.

### Dual-luciferase reporter assay

Potential miRNA-binding sites within the target gene 3′-UTRs were predicted using TargetScan (Release 7.2). Wild-type (WT) and mutant (MUT) DNA fragments were synthesized (Sangon Biotech) and cloned into NheI/AccI-digested pmirGLO vectors using T4 ligase (Takara, Dalian, China), with the sequences listed in Table S3. The recombinant plasmids were transformed into *DH5α* cells, verified, and purified. BMECs were co-transfected with 1.5 μg of WT/MUT plasmids and miR-423-5p/125b mimics. After 48 h, the relative activity normalized to Renilla/firefly luminescence ratios was measured by dual-luciferase activity kit (Promega, Madison, WI, USA).

The binding sites of miRNAs in the 3′-UTR regions of the target genes were predicted using TargetScan. Wild-type and mutant DNA fragments were designed for dual-luciferase reporter assays. All constructs were synthesized by Sangon Biotech (Shanghai, China). The fragments were annealed, ligated into NheI/AccI-digested pmirGLO vectors, and transformed into *DH5α* competent cells. The verified plasmids were extracted using an endotoxin-free kit. For validation, BMECs (70% confluent) were co-transfected with 1.5 μg of WT/mut pmirGLO plasmids and miRNA mimics (miR-423-5p and miR-125b). After 48 h, luciferase activity was measured using LAR II/Stop & Glo reagents (Promega), and the relative activity was calculated as the Renilla-to-firefly luminescence ratio.

### Western blot

Total proteins were extracted from BMECs and milk-derived exosomes using radioimmunoprecipitation assay (RIPA) buffer (Boster, Wuhan, China) according to the manufacturer's specifications. Protein concentrations were determined using the BCA quantitative protein assay kit (Beyotime, Shanghai, China). Proteins were separated by SDS-PAGE (SF12, Affinibody LifeScience Co., Ltd., Wuhan, China) and transferred to PVDF membranes, which were blocked with a sealing solution (AIWB-004, Affinibody LifeScience Co., Ltd., Wuhan, China). The membranes were incubated overnight at 4 °C with anti-CD9, CD81, and TSG101 antibodies (ABclonal, China). This was followed by incubation with secondary antibodies (AS014, ABclonal, Wuhan, China; 1:10,000) for 1.5 h at room temperature in the dark. ECL solution (AIWB-006, Affinibody Life Science Co., Ltd., Wuhan, China) was used for the chemiluminescent detection of protein signals using an imaging system (5200, Tanon Science and Technology Co., Ltd., Shanghai, China).

### Statistical analysis

All experiments were performed in triplicate, and statistical analysis was performed using GraphPad Prism 6 software (GraphPad Software, La Jolla, CA, USA) and one-way analysis of variance (ANOVA) with Dunnett’s multiple comparisons test or Student’s *t*-test. The results are expressed as the mean ± standard error of the mean (SEM), and the values were considered statistically significant at ^*^P < 0.05, ^**^P < 0.01, and ^***^P < 0.001.

## Results

### DHI analysis and grouping with differing MFP

We first performed statistical and correlation analyses on 17,838 DHI records from Chinese Holstein cows to evaluate the effects of lactation stage on milk yield and composition. The results showed a progressive decline in milk yield across the lactation stages, with significant differences (*P* < 0.01) among all periods (Table 1). In contrast, MFP, MPP, and the milk fat-to-protein ratio increased steadily, rising from 3.60% ± 1.19%, 3.12% ± 0.35%, and 1.16% ± 0.39% in early lactation to 4.28% ± 1.05%, 3.50% ± 0.42%, and 1.22% ± 0.27% in terminal lactation, respectively. The 4% FCM yield peaked during late lactation (33.75 ± 10.52 kg/d) but was lowest at mid-lactation, indicating dynamic shifts in the energy-corrected production. SCC increased by 56.6% from early to late lactation, accompanied by an increase in SCS. Interestingly, MUN content was lowest during mid- and late-lactation (17.19 ± 5.26 and 17.27 ± 5.23 mg/dL, respectively) compared with early and terminal stages, suggesting transient reductions in nitrogen utilization efficiency at mid-lactation before recovery in terminal lactation.

**Table 1:** Comparison of milk yield and milk component levels of Chinese Holstein in different lactation periods

Milk quality traits	Lactation stage			
	0–99 d (*n* = 2,035)	100–199 d (*n* = 2,894)	200–299 d (*n* = 1,783)	≥ 300 d (*n* = 924)
Milk yield, kg/d	35.2 ± 8.11^A^	33.8 ± 7.56^B^	32.52 ± 8.2^C^	31.25 ± 10.41^D^
MFP, %	3.6 ± 1.19^A^	3.67 ± 1.08^B^	4.24 ± 1.07^C^	4.28 ± 1.05^C^
4% FCM, kg/d	32.94 ± 9.45^B^	32.05 ± 8.97^Aa^	33.75 ± 10.52^C^	32.63 ± 12.39^ABb^
MPP, %	3.12 ± 0.35^A^	3.26 ± 0.33^B^	3.43 ± 0.36^C^	3.5 ± 0.42^D^
MF/MP	1.16 ± 0.39^B^	1.13 ± 0.33^A^	1.24 ± 0.3^C^	1.22 ± 0.27^C^
MUN content, mg/dL	17.6 ± 5.16^A^	17.19 ± 5.26^B^	17.27 ± 5.23^B^	17.67 ± 5.15^A^
SCC, 10^4^/mL	15.61 ± 43.03^A^	16.44 ± 44.41^A^	20.34 ± 43.28^B^	24.45 ± 46.12^C^
SCS	1.98 ± 1.88^A^	2.4 ± 5.05^B^	2.93 ± 4.37^C^	3.36 ± 4.73^D^

Different lowercase letters in the superscripts indicate a significant difference (*P* < 0.05), and different uppercase letters indicate highly significant differences (*P* < 0.01). *MFP *Milk fat percentage, *MPP *Milk protein percentage, *MF/MP *Milk fat/milk protein, *FCM *Fat-Corrected Milk, *SCC *Somatic cell count, *SCS *Somatic cell scores, *MUN *Milk urea nitrogen

**Fig. 2:**
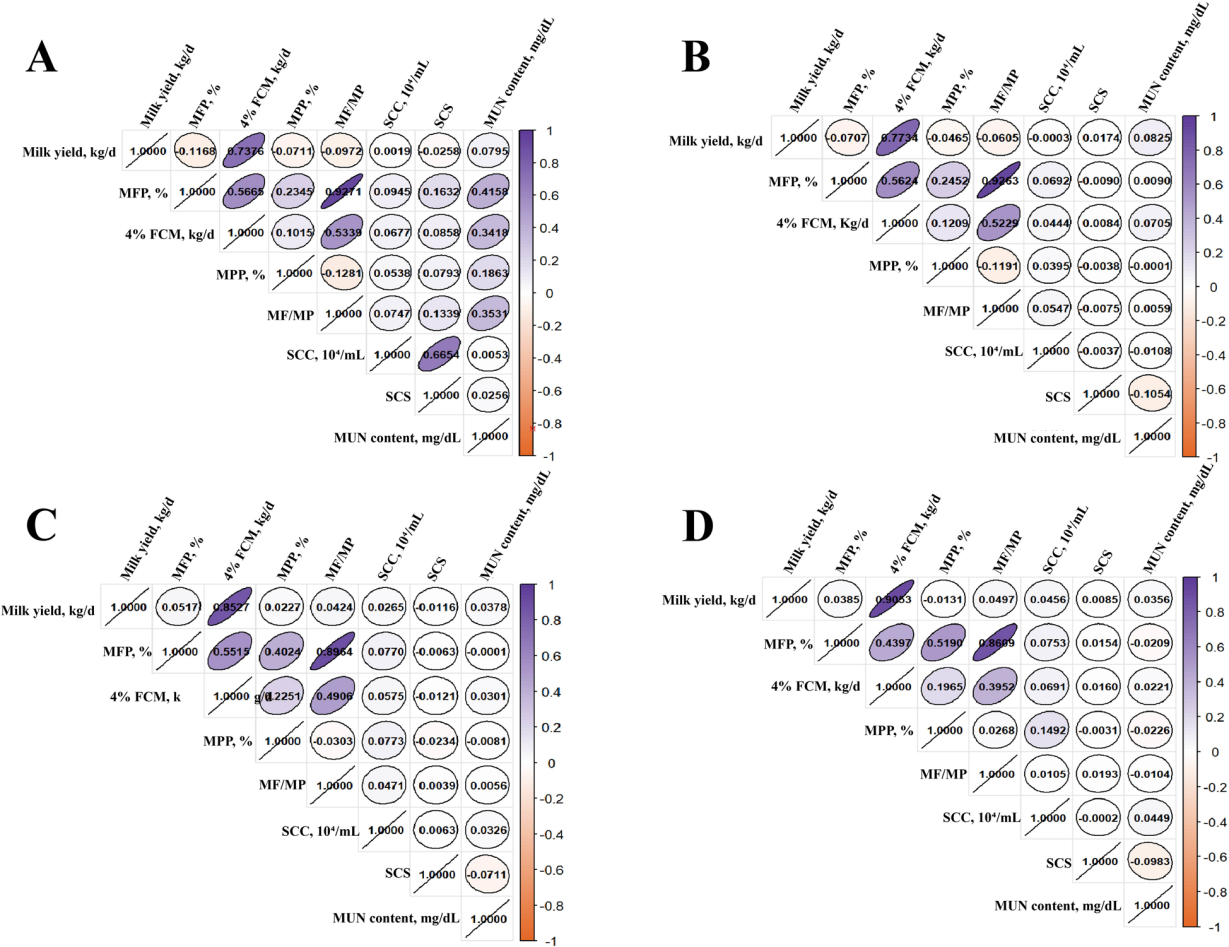
Correlation analysis of milk yield and milk components of Chinese Holstein cattle at different lactation periods. **A** Early lactation (0–99 d). **B** Mid-lactation (100–199 d). **C** Late lactation (200–299 d). **D** Terminal lactation (≥ 300 d). FCM, fat-corrected milk; SCC, somatic cell count; SCS, somatic cell score; MUN, milk urea nitrogen

Correlation analysis further revealed that milk yield was negatively correlated with MFP in early and mid-lactation, whereas 4% FCM remained positively associated with milk yield (Fig. 2). Strong positive correlations between milk fat and protein percentages suggest overlapping metabolic regulation of lipid and protein synthesis in the mammary gland. In late lactation, the milk fat percentage was positively correlated with milk yield, milk protein, and 4% FCM. SCC was positively correlated with milk fat, implying a potential link between lipid metabolism and udder health. MUN showed minimal correlation with other traits, consistent with its role as an independent metabolic marker of energy balance. Based on these findings, second-parity cows in late lactation with SCC < 10^4^ cells/mL were selected as candidates for the high and low milk fat percentage (HMF and LMF) groups, providing representative samples for subsequent exosome and miRNA analyses. The detailed grouping information and corresponding DHI data are presented in Tables S4 and S5.

### Isolation and identification of milk-derived exosomes

**Fig. 3:**
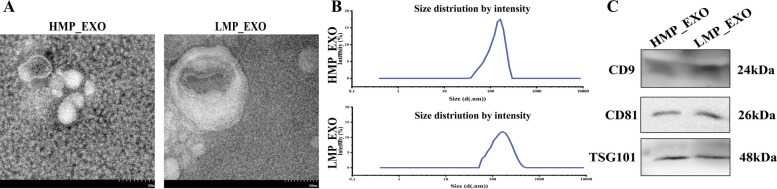
Identification of milk-derived exosomes. **A** Electron microscopy of exosomes. **B** NTA particle size in milk-derived exosomes. **C** Western blotting detection of CD9, CD81, and TSG101 in milk-derived exosomes

After the initial centrifugation of the milk sample, abundant milk proteins, exfoliated mammary epithelial cells, and other impurities settled at the bottom, while a layer of milk fat rose to the top. The middle portion was collected for subsequent ultracentrifugation. During differential ultracentrifugation, the middle two-thirds of the suspension was selected for further processing to minimize contamination with residual milk proteins. Two groups of milk-derived exosomes were obtained using the optimized centrifugation method. Transmission electron microscopy (TEM) revealed spherical vesicles consistent with the characteristic size and morphology of exosomes (HMF_EXO and LMF_EXO, exosomes isolated from HMF and LMF cows, respectively) (Fig. 3A). The vesicles exhibited a typical bilayer membrane structure, with some showing central depressions and a particle diameter of approximately 100 nm. Nanoparticle tracking analysis (NTA) revealed a particle diameter distribution profile with a peak at 100–150 nm (Fig. 3B). Western blot analysis demonstrated the presence of exosomal protein markers CD9, CD81, and TSG101 (24 kDa, 26 kDa, and 48 kDa, respectively; Fig. 3C). These results indicate that milk-derived exosomes were successfully isolated using the optimized procedures and were suitable for subsequent experiments.

### Identification and screening of candidate miRNAs associated with MFP from milk-derived exosomes

**Fig. 4:**
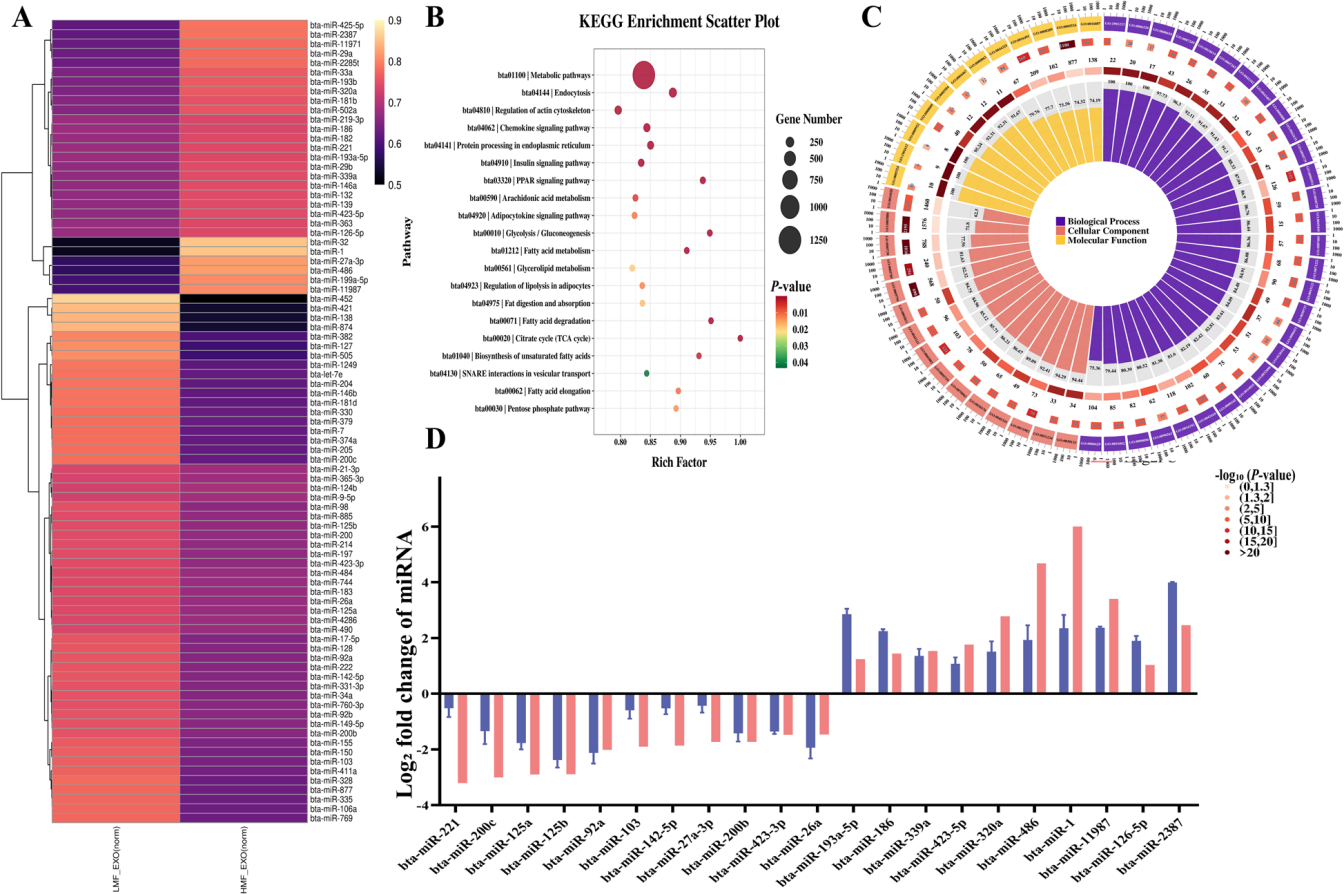
miRNA sequencing and analysis of exosomes derived from milk with high and low milk fat percentages. **A** Heatmap of differentially expressed miRNA clusters. **B** KEGG enrichment analysis of differentially expressed miRNAs. **C** GO enrichment analysis of differentially expressed miRNAs. **D** Validation of differentially expressed miRNAs using qRT-PCR

Exosomal miRNA sequencing libraries were constructed to identify DEmiRNAs between HMF_EXO and LMF_EXO. After quality control and adapter trimming, 20,003,230 and 18,982,930 clean reads were obtained from the HMF_EXO and LMF_EXO groups, respectively, with Q20 values exceeding 99% in both groups (Table S5). Most of the identified miRNAs were 22–29 nt in length (Fig. S2). Differential expression analysis revealed 1,320 DEmiRNAs between the two groups, including 496 upregulated and 824 downregulated miRNAs (Fig. S3). Highly expressed miRNAs were predominantly enriched in metabolic pathways, PPAR signaling pathways, and other lipid metabolism-relevant pathways. The top 100 DEmiRNAs (ranked by *P*-value) are displayed as a heatmap (Fig. 4A). GO and KEGG enrichment analyses of the target genes (Fig. 4B and C) showed significant enrichment in key metabolic and signal transduction pathways, including the metabolic and PPAR signaling pathways. Twenty-one miRNAs exhibited consistent expression patterns between the RNA-seq and qRT-PCR validation results (Fig. 4D), confirming the sequencing data reliability.

### Exosome uptake and screening of candidate miRNAs

**Fig. 5:**
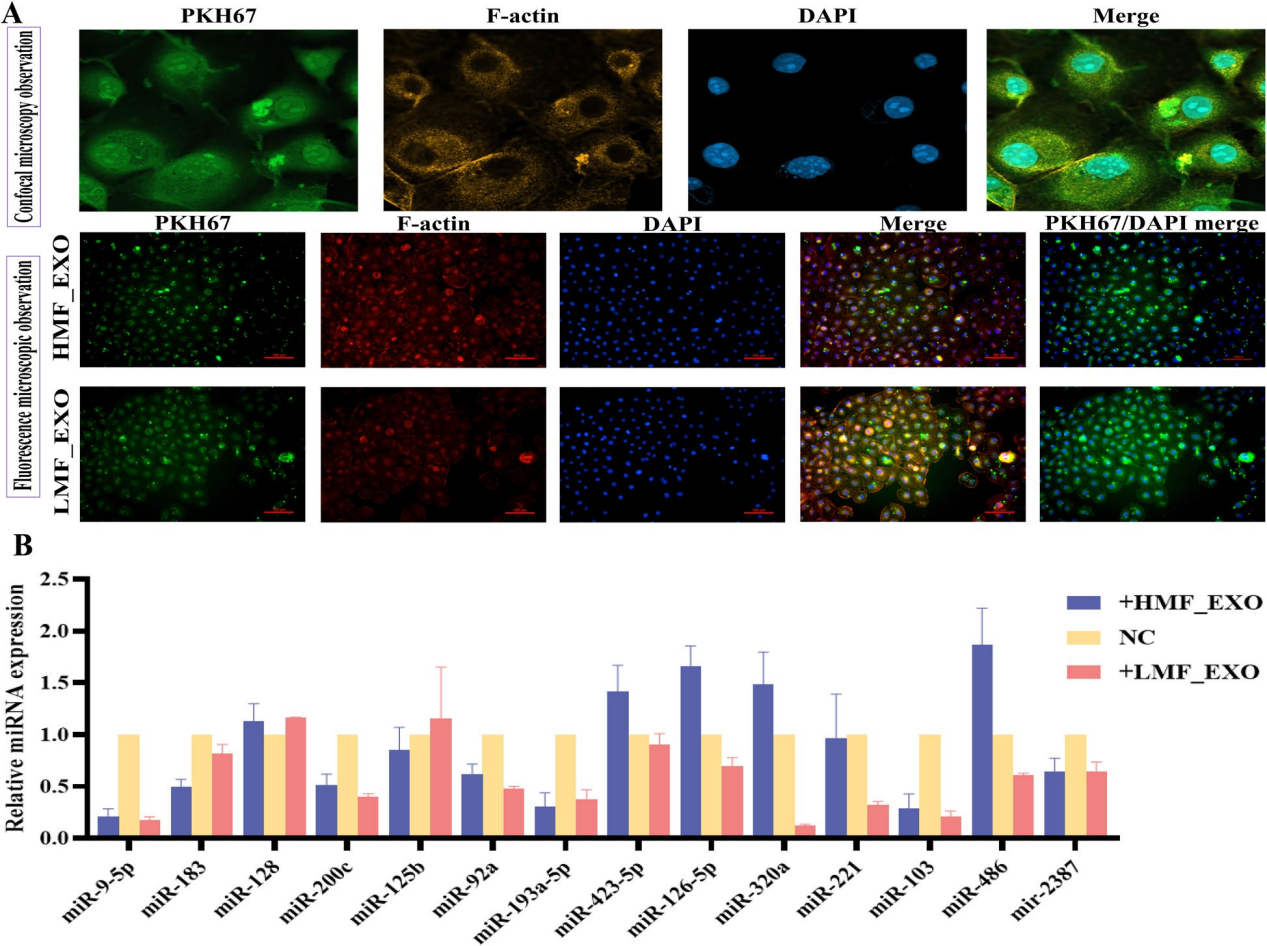
Exosome uptake and functional validation of candidate miRNAs. **A** Uptake of milk-derived exosomes by BMECs. **B** Expression of miRNAs in BMECs following the addition of HMF- and LMF milk-derived exosomes

After co-culturing the cells with fluorescently labeled exosomes, green fluorescence was detected using confocal and fluorescence microscopes, indicating that exosomes were internalized with genetic material into BMECs through endocytosis or similar processes (Fig. 5A). Following treatment of BMECs with exosomes isolated from the HMF_EXO and LMF_EXO groups, miR-126-5p, miR-320a, and miR-423-5p showed higher expression levels in HMF_EXO-treated BMECs, whereas miR-125b showed the highest expression in the LMF_EXO group (Fig. 5B). However, preliminary functional validation experiments indicated that miR-320a had no significant effect on lipid metabolism int these cells. Based on miRNA profiling and functional validation, miR-423-5p and miR-125b were selected as candidate miRNAs for subsequent analysis.

### Milk-derived exosomal miR-423-5p/*APOA5* enhanced lipid metabolism in BMECs

**Fig. 6:**
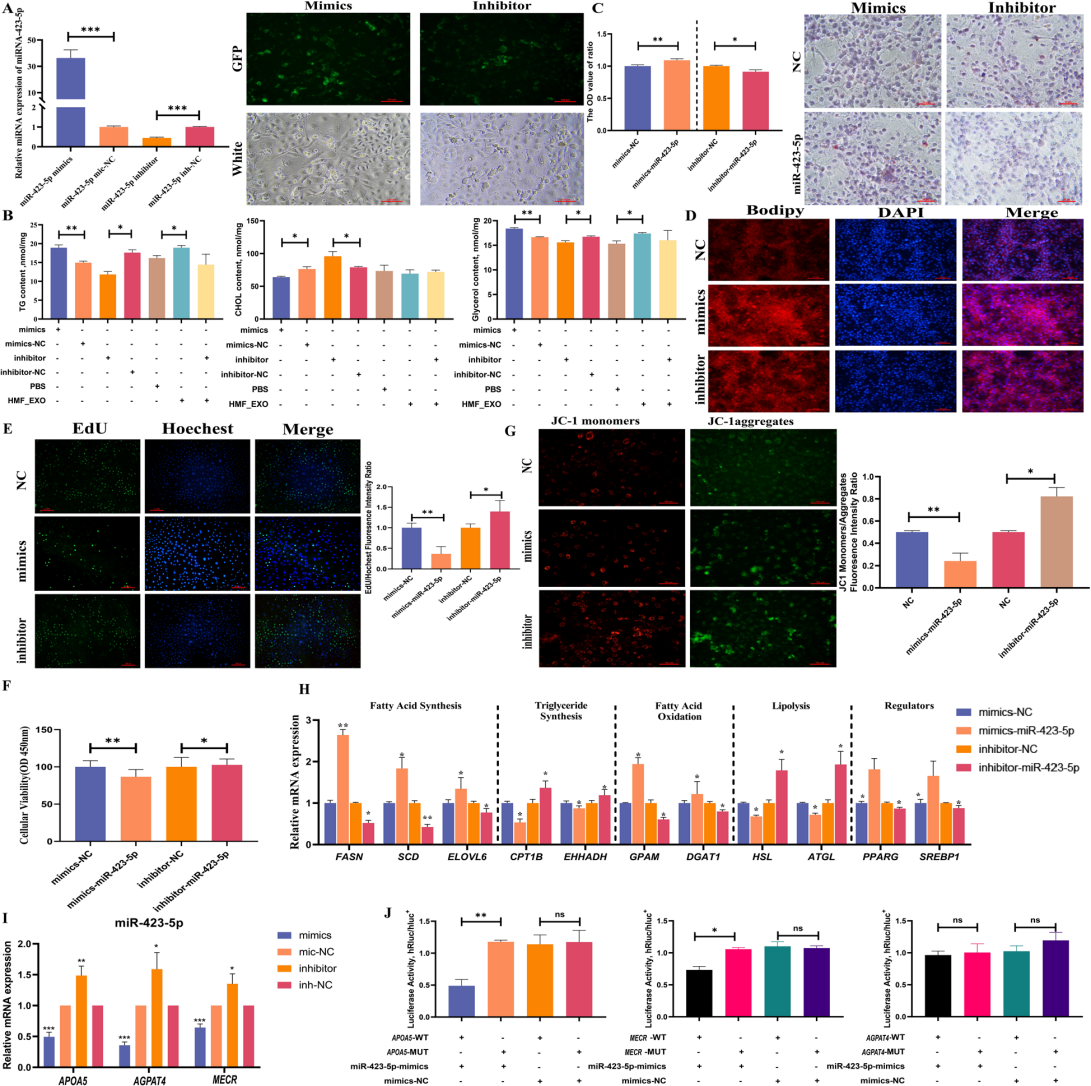
Milk-derived exosomal miR-423-5p enhances lipid metabolism in BMECs. **A** Validation of the transfection efficiency of miR-423-5p mimics and inhibitors. **B** Effects of exosomal miR-423-5p on glycerol, TG, and CHOL contents in BMECs. **C** and **D** Effects of miR-423-5p on lipid droplets in BMECs detected by (**C**) Oil Red O and (**D**) BODIPY staining. **E** Effect of miR-423-5p on the proliferation of BMECs as determined by EdU assay. **F** Effect of miR-423-5p on the cellular viability of BMECs of CCK8. **G** Effect of miR-423-5p on the membrane potential of BMECs. **H** Effects of miR-423-5p on genes related to lipid metabolism in BMECs. **I** Effects of miR-423-5p on the mRNA expression levels of candidate target genes. **J** Verification of the target relationship between miR-423-5p and *APOA5*. ^***^P < 0.001, ^**^P < 0.01, ^*^P < 0.05, ns: not significant

The transfection efficiency of miR-423-5p and its effect on intracellular lipid content were verified. Compared to mimics-NC, the miR-423-5p mimics significantly upregulated target expression (*P* < 0.001), whereas the inhibitors downregulated expression compared to inhibitor-NC (*P* < 0.001, Fig. 6A). MiR-423-5p overexpression promoted the intracellular accumulation of glycerol and TG (*P* < 0.01) while reducing CHOL content (*P* < 0.05). To exclude interference from endogenous miRNAs, the miR-423-5p inhibitor was delivered via exosomes for validation. HMF_EXOs treatment reproduced the TG content induced by miR-423-5p mimics (*P* < 0.05), with a similar trend observed in CHOL content, while glycerol content also increased significantly (*P* < 0.05). The inhibitor-loaded HMF_EXO showed no difference from the PBS group. These findings confirmed that exosomal delivery abolished the regulatory effects of miR-423-5p on lipid metabolism (Fig. 6B).

The effects of miR-423-5p on intracellular lipid droplets, cell proliferation, membrane potential, and lipid metabolism-related genes were further examined. The miR-423-5p mimics enhanced intracellular lipid droplet accumulation (*P* < 0.01), whereas its inhibitor reduced lipid droplet formation (*P* < 0.05), consistent with Oil Red O and BODIPY staining results (Fig. 6C and D). Furthermore, miR-423-5p mimics significantly reduced the proliferation and viability of BMECs compared to the mimics-NC group (*P* < 0.01), whereas the inhibitor enhanced proliferative activity and viability (*P* < 0.05, Fig. 6E and F). Transfection with miR-423-5p mimics significantly decreased membrane potential (*P* < 0.01), and the inhibitors further reduced it, as determined by JC-1 ratio measurement (*P* < 0.05, Fig. 6G). MiR-423-5p mimics significantly upregulated the expression of lipogenic genes (*FASN*, *SREBP1*) and TG synthesis-related genes (*DGAT1*, *GPAM, P* < 0.05) while downregulating the fatty acid oxidation marker *CPT1B* (*P* < 0.01, Fig. 6H), indicating a role of miR-423-5p in stimulative lipid synthesis and suppressive oxidative remodeling by qRT-PCR analysis.

The target relationship between miR-423-5p and *APOA5* was validated. The predicted lipid metabolism-related targets of miR-423-5p included *APOA5*, *AGPAT4*, and *MECR*. Transfection with the miR-423-5p mimics significantly reduced mRNA expression levels of *APOA5*, *AGPAT4*, and *MECR*, whereas transfection with its inhibitor elevated their expression (Fig. 6I). The miR-423-5p mimics markedly decreased luciferase activity in wild-type *APOA5* (*P* < 0.01) and *MECR* (*P* < 0.05) vectors, whereas no significant alterations were observed in their mutant-type constructs or negative control groups, as revealed by dual-luciferase reporter assays (Fig. 6J). Based on these results, *APOA5* was identified as the functional target gene of miR-423-5p that regulates lipid metabolism.

**Fig. 7:**
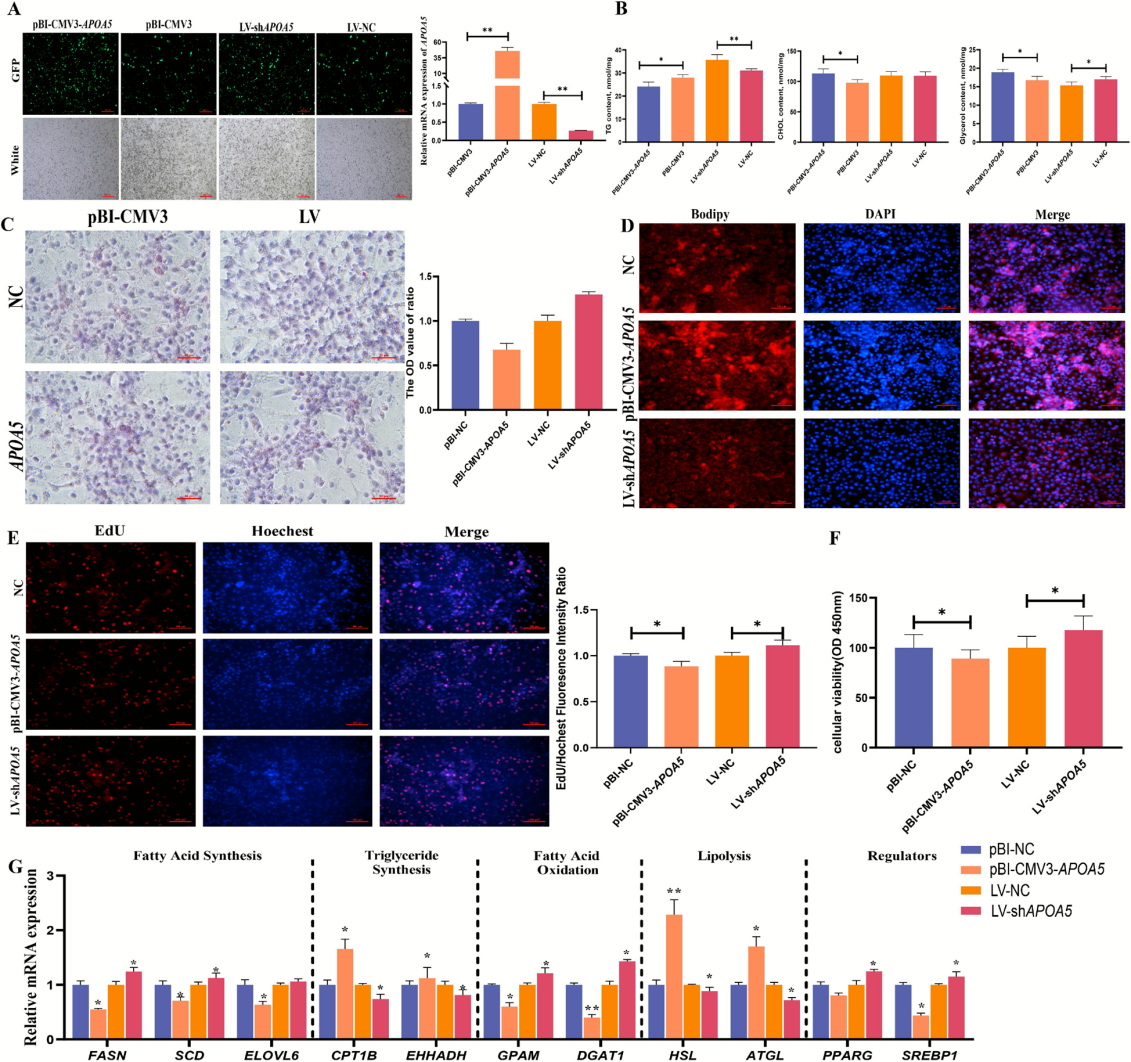
Effect of target gene *APOA5* on lipid metabolism in BMECs. **A** Validation of pBI-CMV3-*APOA5* and LV-sh*APOA5* vector efficiencies. **B** Effects of *APOA5* on glycerol, TG, and CHOL contents in BMECs. **C** and **D** Effects of *APOA5* on lipid droplets in BMECs were detected by (**C**) Oil Red O and (**D**) BODIPY staining. **E** The effect of *APOA5* on the proliferation of BMECs, as measured by EdU. **F** Effect of *APOA5* on the cellular viability of BMECs of CCK8. **G** Effect of *APOA5* on lipid metabolism-related genes in BMECs.^**^P < 0.01, ^*^P < 0.05

The impact of the target gene *APOA5* on lipid metabolism was further validated. BMECs were transfected with the overexpression vector pBI-CMV3-*APOA5* and the knockdown construct LV-sh*APOA5*, respectively. At 24 h post-transfection, the mRNA expression of *APOA5* was significantly upregulated in pBI-CMV3-*APOA5* and downregulated in LV-sh*APOA5* (*P* < 0.01, Fig. 7A)*.* The overexpression of *APOA5* reduced TG content while increasing CHOL and glycerol concentrations in BMECs (*P* < 0.05). Conversely, *APOA5* knockdown increased TG (*P* < 0.01) and decreased glycerol levels (*P* < 0.05), with no change in CHOL content (Fig. 7B). *APOA5* overexpression significantly reduced intracellular lipid droplets (*P* < 0.01), whereas its knockdown induced their formation (*P* < 0.01), as confirmed by BODIPY staining (Fig. 7C and D). Furthermore, *APOA5* overexpression impaired cellular viability (*P* < 0.05) and reduced the EdU cell population (*P* < 0.05), whereas its knockdown increased cell viability (*P* < 0.05), indicating that *APOA5* acts as a negative regulator of BMEC proliferation (Fig. 7E and F). *APOA5* overexpression significantly downregulated key fatty acid synthesis genes, including *FASN*, *SCD*, and *ELOVL6*, while upregulated fatty acid oxidation genes (*CPT1B* and *EHHADH*,* P* < 0.05, Fig. 7G).

### Milk-derived exosomal miR-125b/*SLC27A1* suppressed lipid metabolism in BMECs

**Fig. 8:**
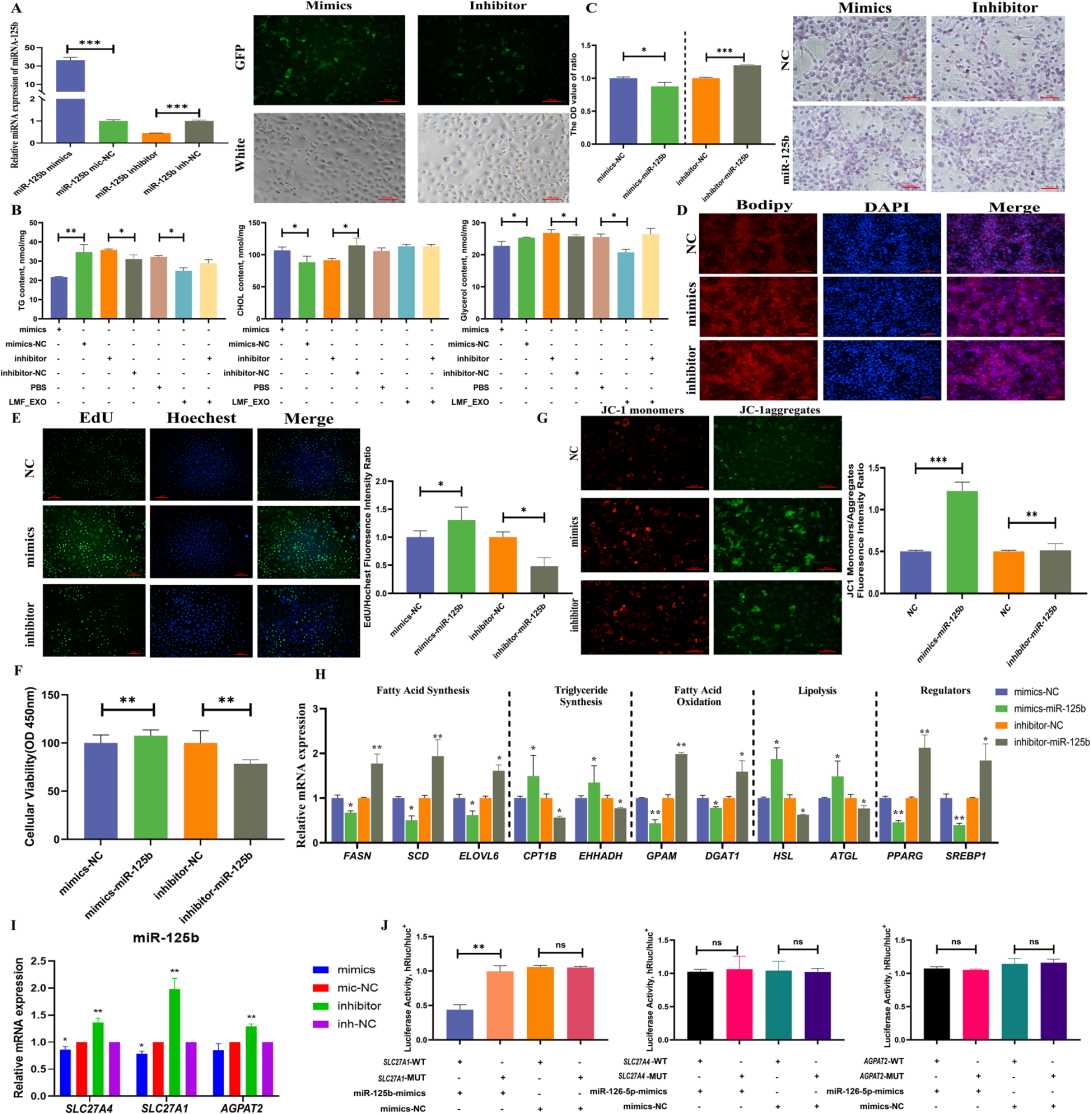
Milk-derived exosomal miR-125b suppresses lipid metabolism in BMECs. **A** Validation of the transfection efficiency of miR-125b mimics and inhibitors. **B** Effects of exosomal miR-125b on glycerol, TG, and CHOL contents in BMECs. **C** and **D** Effects of miR-125b on lipid droplets in BMECs were detected by (**C**) Oil Red O and (**D**) BODIPY staining. **E** Effect of miR-125b on the proliferation of BMECs as determined by EdU assay. **F** Effect of miR-125b on the cellular viability of BMECs of CCK8. **G** Effect of miR-125b on membrane potential in BMECs. **H** Effects of miR-125b on genes related to lipid metabolism in BMECs. **I** Effects of miR-125b on the mRNA expression levels of candidate target genes. **J** Verification of the target relationship between miR-125b and *SLC27A1*.^***^P < 0.001, ^**^P < 0.01, ^*^P < 0.05, ns: not significant

The transfection efficiency of miR-125b and its effect on the intracellular lipid content were verified. Transfection with miR-125b mimics significantly increased miR-125b expression, whereas its inhibitor decreased expression compared to that in the NC group (*P* < 0.05, Fig. 8A). Elevated miR-125b augmented intracellular glycerol and TG content, but diminished CHOL content. To circumvent endogenous miRNA interference, exosome-mediated delivery of the miR-125b inhibitor was employed. Treatment with LMF_EXOs reproduced the TG increase induced by miR-125b mimics (*P* < 0.05), with a similar trend for CHOL and a significant increase in glycerol (*P* < 0.05, Fig. 8B). Inhibitor-loaded LMF_EXO exhibited no divergence from the PBS control group, verifying that exosomal delivery abolished the regulatory effects of miR-125b on lipid metabolism.

The effects of miR-125b on lipid metabolism were also investigated. Oil Red O and BODIPY staining showed that miR-125b mimics enhanced lipid droplet deposition, whereas the inhibitor inhibited droplet formation (*P* < 0.05, Fig. 8C and D). EdU incorporation and CCK-8 assays were systematically performed to evaluate the effects of miR-125b on the proliferation and viability of cells. The results showed that miR-125b mimics promoted proliferative activity (*P* < 0.05), whereas the inhibitors suppressed it (*P* < 0.05, Fig. 8E and F). Transfection with miR-125b mimics significantly decreased the mitochondrial membrane potential (*P* < 0.001), while its inhibitor increased it (*P* < 0.01, Fig. 8G). MiR-125b mimics markedly reduced the expression of lipolysis-related genes (*HSL* and *ATGL*, *P* < 0.05) and the transcriptional regulator *PPARG* (*P* < 0.01), suggesting its role in facilitating lipid mobilization (Fig. 8H). Collectively, these results indicate that miR-125b exerts an antagonistic effect on lipid synthesis and catabolism in BMECs.

The target relationship between miR-125b and *SCL27A1* was validated. Predicted lipid metabolism–related targets of miR-125b (*SLC27A1*, *SLC27A4* and *AGPAT2*) showed diminished mRNA abundance after mimic transfection but increased expression following inhibitor treatment (Fig. 8I). MiR-125b mimics significantly repressed the luciferase activity of wild-type *SLC27A1* reporters (*P* < 0.05), while no significant changes were observed in mutant constructs or negative controls (Fig. 8J). These results indicate that *SLC27A1* was identified as a functional target of miR-125b and played a pivotal role in lipid metabolism in BMECs.

**Fig. 9:**
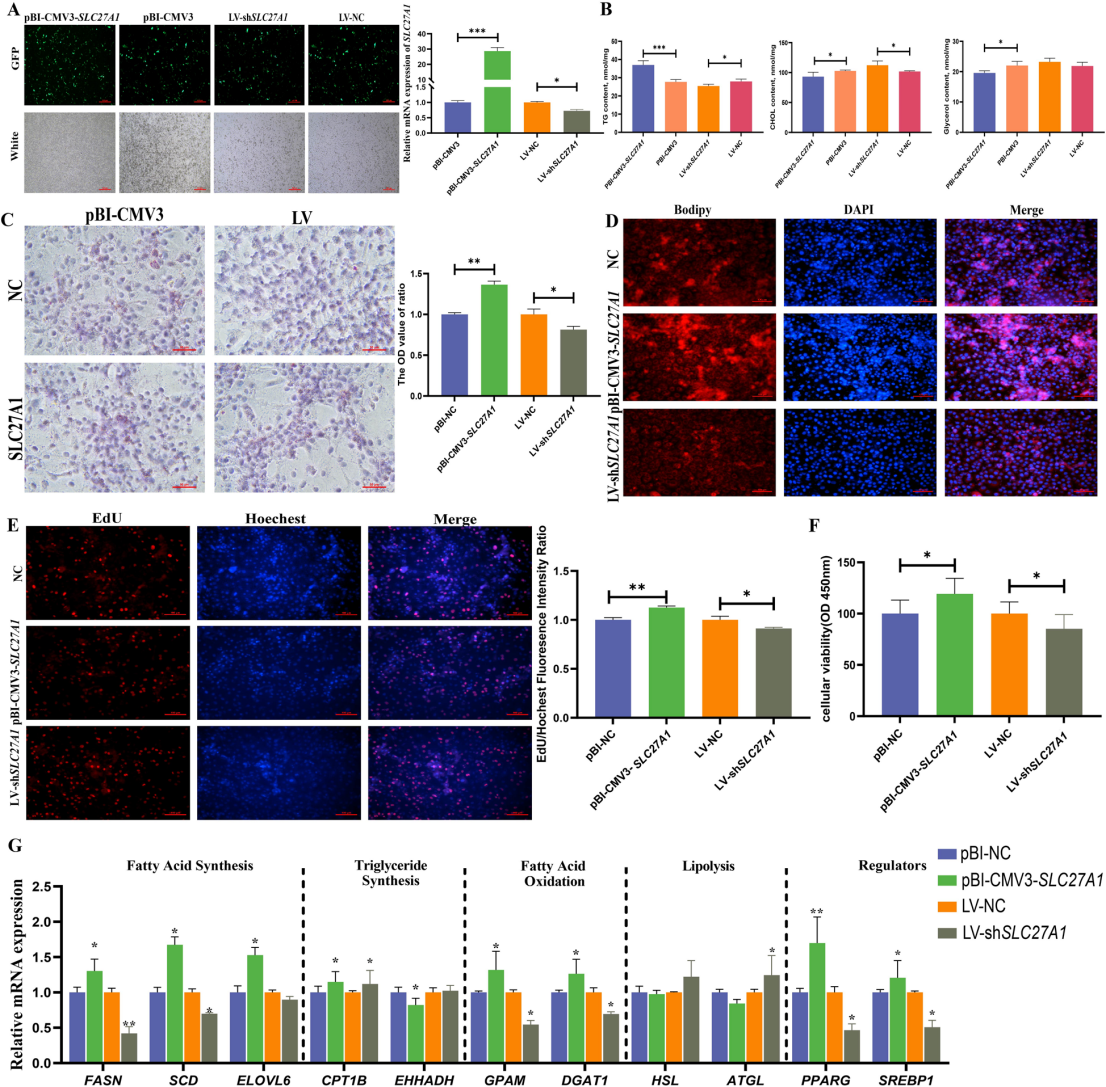
Effect of target gene *SLC27A1* on lipid metabolism in BMECs. **A** Validation of pBI-CMV3-*SLC27A1* and LV-sh*SLC27A1* vector efficiencies. **B** Effects of *SLC27A1* on glycerol, TG, and CHOL contents in BMECs. **C** and **D** Effects of *SLC27A1* on lipid droplets in BMECs detected by (**C**) Oil Red O and (**D**) BODIPY staining. **E** Effect of *SLC27A1* on the proliferation of BMECs of EdU. **F** Effect of *SLC27A1* on the cellular viability of BMECs of CCK8. **G** Effect of *SLC27A1* on lipid metabolism-related genes in BMECs.^***^P < 0.001, ^**^P < 0.01, ^*^P < 0.05

The regulatory role of the target gene *SCL27A1* in lipid metabolism was also validated. BMECs transfected with the overexpression vector (pBI-CMV3-*SLC27A1*) or knockdown construct (LV-sh*SLC27A1*) exhibited corresponding changes in *SLC27A1* mRNA expression (*P* < 0.001, *P* < 0.05, Fig. 9A). *SLC27A1* overexpression depressed TG content (*P* < 0.05) and elevated CHOL and glycerol content (*P* < 0.05). Conversely, *SLC27A1* knockdown amplified TG content (*P* < 0.01) and reduced glycerol levels, while leaving CHOL content unchanged (Fig. 9B). Lipid droplet accumulation was attenuated by overexpression (*P* < 0.01) and potentiated by knockdown (*P* < 0.05), consistent with BODIPY quantification (Fig. 9C and D). Furthermore, *SLC27A1* overexpression compromised cell viability (*P* < 0.01) and reduced the number of EdU-positive cells, whereas knockdown enhanced cell viability (*P* < 0.05), establishing *SLC27A1* acted as a negative regulator of BMEC proliferation (Fig. 9E and F). *SLC27A1* overexpression upregulated lipogenic genes (*FASN*, *SCD* and *ELOVL6*, *P* < 0.05) and downregulated β-oxidation genes (*CPT1B* and *EHHADH*, *P* < 0.05, Fig. 9G).

## Discussion

The mammary gland functions not only as a secretory organ, but also as a highly sophisticated regulatory system. During milk synthesis, BMECs, adipocytes, and immune cells [42] actively secrete exosomes, nanoscale vesicles that act as molecular messengers. These exosomes carry a variety of genetc materials, including miRNAs, which serve as feedback signals to precisely modulate the functional activities of neighboring or self-MECs [39, 43]. This regulatory mechanism contributes to the optimization of milk production, particularly in energy-intensive lipid metabolic pathways. To validate the regulatory role of exosomes, this study conducted in vivo experiments by reintroducing milk-derived exosomes into the cultured BMECs. The exosome treatment significantly altered the expression of genes related to lipid metabolism and enhanced lipid droplet formation, strongly supporting the existence of an autoregulatory mechanism within the mammary gland. Primiparous (first-lactation) cows are typically still maturing, and their milk production is often lower and more variable, whereas older cows (third parity and beyond) may be more susceptible to health issues like mastitis, which can confound the results [44]. By focusing on second-parity cows, we aimed to minimize these non-genetic sources of variation, thereby increasing the likelihood that the observed differences in milk fat percentage could be attributed to the genetic and molecular mechanisms under investigation. This sample size was determined based on an extreme phenotype selection strategy from a large population to maximize the detection of molecular differences associated with the milk fat percentage.

The primary goal of selecting high-yielding dairy cows is not only to enhance milk yield quantitatively but also to produce milk with superior economic value and nutritional quality. In this context, milk fat percentage and milk protein percentage, as critical lactation traits, are of comparable importance to the total milk production. In the process of selecting high-yielding dairy cows, placing milk fat percentage and milk protein percentage on par with or even above the total milk yield is an essential strategy to improve the economic efficiency of dairy farming, fulfill the requirements of the dairy processing industry, and align with the preferences of end consumers. In the present investigation, the analysis of DHI data revealed a significant positive correlation between milk fat content and lactation stage, consistent with relevant research on lipid metabolism during mid-lactation [45]. Notably, a 56.6% increase in SCC and elevated milk fat percentage was correlated (*r* = 0.71) during late lactation. This result suggests that milk fat synthesis may modulate mammary inflammation by altering the activity of enzymes implicated in the immune response [16]. Although the fat percentage of milk reaches its peak at the last lactation, to minimize the confounding effects of inflammation, this study specifically selected last-lactation cows (200–299 d) as experimental subjects, thereby establishing a stable sample for investigating the genetic determinants of variation in milk fat.

The protein and lipid contents in milk were higher than those in cell culture medium. To avoid the interference of a large amount of protein on the purity of exosomes, this study established an optimized protocol that combines differential centrifugation with 0.22 μm filtration to obtain high-purity exosomes of the appropriate particle size. The initial centrifugation stage employed gradient defatting to remove large-diameter (> 1 μm) milk fat globular membrane fragments. In recent years, newer methods for milk exosome isolation have become available, such as size-exclusion chromatography (SEC) [46], which is capable of preserving the structural integrity of exosomes owing to its gentle separation mechanism, thereby minimizing potential damage to their biological functions. Chemical precipitation methods (such as the PEG method) achieve rapid enrichment by competitive binding of polymers to hydrophilic groups on the surface of exosomes. Although they are simple to operate and inexpensive, the accompanying contamination of impurity proteins (contamination rate > 30%) may affect the accuracy of downstream analysis [47]. Considering the experimental requirements for cell treatment procedures in this study, we opted for traditional differential ultracentrifugation despite its limitations in ensuring both the purity and yield of exosomes, which are critical for subsequent functional genomics studies of exosomal genetic material. PKH67-labeled exosomes were predominantly localized in the cytoplasm and cell membrane of BMECs, consistent with previous studies on cell-derived exosomes, which suggested the cellular uptake of milk-derived exosomes via endocytic pathways [48]. Exosomal lipid protection may explain the efficient delivery of miRNAs observed in future research. Furthermore, milk-derived exosomes have the potential to efficiently and conveniently manipulate exosomal miRNAs, enabling the successful delivery of exosomal miRNA molecules to recipient cells [49]. Exosomal contents can interact with cytoplasmic target molecules. This represents a potential mechanism by which exosomes mediate intercellular communication and modulate key cellular processes. However, it is crucial to recognize that these exosomal effects occur in concert with the recipient cell’s intrinsic regulatory networks, ultimately influencing its physiological state, gene expression, and fate decisions, such as proliferation, differentiation, or apoptosis.

Exosomal miRNA sequencing identified 1,320 DEmiRNAs, with miR-423-5p and miR-125b showing specific enrichment in the high- and low-milk-fat groups, respectively. KEGG enrichment analysis revealed that the target genes of these miRNAs were significantly clustered in PPAR signaling pathway and fatty acid elongation pathway. miRNA sequencing analysis demonstrated that the expression of miR-423-5p in exosomes from the high-fat milk group was significantly elevated. The uptake of the HMF_EXO group by BMECs could lead to a similar promoting effect on TG content as the mimics of miR-423-5p. However, the intensity of the promoting effect was not as strong as that of exogenously synthesized mimics. However, this indicates that the milk-derived exosomes in the HMF_EXO group were rich in miRNAs and could serve as biomarkers for lipid metabolism. Meanwhile, this study also explored the inhibitory effect of miR-125b on TG content. After the addition of LMF_EXOs, the TG content in BMECs showed a downward trend. Further experiments indicated that co-incubation of the miR-125b inhibitor with LMF_EXOs and then adding a mixture to BEMCs resulted in a rescue effect. Previous studies have conducted a combined analysis of RNA-seq and miRNA-seq derived from RNAs extracted from BMECs with high and low milk fat percentage [41]. This approach was designed to identify candidate DEmiRNAs and their target genes associated with the milk fat percentage. We further compared the DEmiRNAs identified in our analysis with those derived from milk exosomes and identified 168 overlapping DEmiRNAs. Notably, miR-423-5p and miR-125b, two key candidates identified in this study, were included in this subset. In addition, the common DEmiRNAs identified through joint screening, including miR-30c [50], miR-200 [51], and miR-34 [52], have been well documented to play regulatory roles in lipid metabolism. The distinct differential miRNA profiles observed in BMECs versus milk-derived exosomes suggest that miRNAs in exosomes are not exclusively derived from BMECs, and that BMECs may selectively package specific miRNAs into exosomes to regulate other cell types within mammary tissue. Furthermore, these comparative results support our hypothesis that cows with divergent milk fat phenotypes produce exosomes with distinct miRNA cargos, which in turn differentially regulate the metabolic activity of mammary cells.

Various cell types in the mammary gland modulate milk fat synthesis in BMECs and affect breast health via exosomal secretion and transfer. This study systematically elucidated the molecular mechanism by which milk-derived exosomal miR-423-5p and miR-125b regulate lipid metabolism via the *APOA5/SLC27A1-SREBP1/PPARγ* network. The validation results in BMECs revealed that *APOA5* overexpression markedly inhibited *SREBP1* and *PPARγ* expression, consistent with the central role of the *SREBP1-PPARγ* axis in regulating lipid metabolism. However, this study further elucidated that *APOA5* promotes fatty acid oxidation by upregulating *CPT1B* expression. In contrast, interference with *SLC27A1* increased *PPARγ* expression, thereby promoting TG synthesis via activation of *GPAM* and *DGAT1*, which parallels the *AGPAT6* action pathway but emphasizes the fatty acid uptake stage. Elevated mitochondrial membrane potential (*ΔΨm*) correlates with enhanced mitochondrial bioenergetic activity, whereas a decline reflects depolarization and functional impairment [53]. This perturbation compromises ATP synthesis efficiency and disrupts the TG biosynthetic pathway, particularly the esterification of fatty acids with glycerol, catalyzed by diacylglycerol acyltransferase (*DGAT*) [54]. Collectively, these findings delineate an intricate regulatory network in BMECs through exosomes, where miRNAs target the *APOA5* gene and *SLC27A1* exerts opposing effects on lipid metabolism, a process fundamentally constrained by mitochondrial bioenergetic status. This study provides a novel mechanistic framework for understanding intracellular lipid homeostasis. This study was limited to verifying the effects of exosomes on BMECs and the mechanisms of select miRNAs within a single in vitro model. Consequently, the impact on other mammary cell types, including adipocytes, remains to be further investigated.

This study hypothesized that there are significant differences in the molecular profiles of exosomes in the milk of dairy cows with high- and low-fat percentage. Specifically, high-quality dairy cows may produce functionally distinct, high-quality messengers that actively contribute to superior milk traits. These results provide novel insights into key functional vehicles and regulators involved in milk lipid synthesis and regulation. Not only does this deepen our theoretical understanding of milk fat synthesis regulation, but it also provides a foundation for the early prediction of milk quality based on exosomal miRNA markers and targeted improvement of milk components via miRNA expression regulation. Incorporating exosomes as functional biomarkers into genotype-assisted selection strategies can significantly improve the accuracy and efficiency of breeding decisions. The potential of using these miRNAs from blood samples of young calves to predict future milk quality is an intriguing possibility. However, this approach requires further validation to confirm the presence and predictive power of these miRNAs in circulation before any application in breeding programs can be considered.

## Conclusions

This study identified milk-derived exosomal miRNAs as key regulators of milk fat synthesis through the miR-423-5p/*APOA5* and miR-125b/*SLC27A1* axes. These findings provide critical insights into the molecular determinants of milk fat composition and reveal the pivotal role of intercellular communication in the mammary gland.

## Supplementary Information

Additional file 1: Table S1. qRT-PCR primer of miRNA and genes related to lipid metabolism. Table S2. The sequence of shRNA. Table S3. Dual-luciferase reporter vector DNA sequences of candidate target genes. Table S4. Summary of DHI data for candidate Holstein cows. Table S5. Detailed DHI data for candidate Holstein cows. Table S6. The table of sample data quality control. Fig. S1. Validation of interference efficiency of LV-shAPOA5/shSLC27A1 vector.

## Data Availability

The datasets produced or analyzed during the current study are available from the corresponding author on reasonable request.

## Abbreviations

ACACA: Acetyl-CoA carboxylase alpha
AGPAT2: 1-Acylglycerol-3-phosphate O-acyltransferase 2
AGPAT4: 1-Acylglycerol-3-phosphate O-acyltransferase 4
AGPAT6: 1-Acylglycerol-3-phosphate O-acyltransferase 6
AMPK: AMP-activated protein kinase
APOA5: Apolipoprotein A5
BMECs: Bovine mammary epithelial cells
CCK8: Cell Counting Kit-8
CHOL: Cholesterol
CPT1B: Carnitine palmitoyltransferase 1B
DEmiRNAs: Differentially expressed miRNAs
DGAT: Diacylglycerol O-acyltransferase
DHI: Dairy herd improvement
EHHADH: Enoyl-CoA hydratase and 3-hydroxyacyl CoA dehydrogenase
ELOVL6: ELOVL fatty acid elongase 6
FASN: Fatty acid synthase
FCM: Fat-corrected milk
GPAM: Glycerol-3-phosphate acyltransferase
HSL: Hormone-sensitive triglyceride lipase
HMF: High milk fat percentage
HMF_EXO: Exosomes derived from high milk fat percentage cows
LMF: Low milk fat percentage
LMF_EXO: Exosomes derived from low milk fat percentage cows
LPIN1: Lipin 1
MECR: Mitochondrial trans-2-enoyl-CoA reductase
MFP: Milk fat percentage
MPP: Milk protein percentage
mTOR: Mechanistic target of rapamycin
MUN: Milk urea nitrogen
PPARG: Peroxisome proliferator-activated receptor gamma
qRT-PCR: Quantitative real-time PCR
SCC: Somatic cell count
SCD: Stearoyl-CoA desaturase
SCS: Somatic cell scores
SLC27A1: Solute carrier family 27 member 1
SLC27A4: Solute carrier family 27 member 4
SREBP: Sterol regulatory element-binding protein
SREBP1: Sterol regulatory element-binding protein 1
TDG: Thymine DNA glycosylase
TG: Triglyceride
VLDLR: Very low-density lipoprotein receptor
ΔΨm: Mitochondrial membrane potential
