# Supplementary material for: Functional analysis and identification of miRNAs associated with lipid metabolism from milk-derived exosomes
Source: J Anim Sci Biotechnol. 2026 Feb 4;17:20. doi: 10.1186/s40104-025-01331-5 (PMC12870503; doi:10.1186/s40104-025-01331-5)
Supplement: Supplementary file 1 — Additional file 1: Table S1. qRT-PCR primer of miRNA and genes related to lipid metabolism. Table S2. The sequence of shRNA. Table S3. Dual-luciferase reporter vector DNA sequences of candidate target genes. Table S4. Summary of DHI data for candidate Holstein cows. Table S5. Detailed DHI data for candidate Holstein cows. Table S6. The table of sample data quality control. Fig. S1. Validation of interference efficiency of LV-shAPOA5/shSLC27A1 vector. [file 40104_2025_1331_MOESM1_ESM.docx]

**Tables**

**Table S1** qRT-PCR primer of miRNA and genes related to lipid metabolism

| **Primers** | **Forward sequences (5´→3´)** | **Reverse sequences (5´→3´)** |
| --- | --- | --- |
| bta-miR-u6 | CACGCAAATTCGTGAAGCGTTCC | |
| miR-423-5p | GGGGCAGAGAGCGAGACTTT | |
| miR-125b | TCCCTGAGACCCTAACTTGTGA | |
| bta-miR-9-5p | GCTCTTTGGTTATCTAGCTGTATGA | |
| bta-miR-183 | ATGGCACTGGTAGAATTCACTG | |
| bta-miR-128 | TCACAGTGAACCGGTCTCTTT | |
| bta-miR-21-3p | CAACACCAGTCGATGGGCTGTC | |
| bta-miR-146a | TGAGAACTGAATTCCATGGGTT | |
| bta-miR-200c | TAATACTGCCGGGTAATGATGGA | |
| bta-miR-125a | TCCCTGAGACCCTTTAACCTGT | |
| bta-miR-125b | TCCCTGAGACCCTAACTTGTGA | |
| bta-miR-92a | TATTGCACTTGTCCCGGCCT | |
| bta-miR-103 | AGCAGCATTGTACAGGGCTATG | |
| bta-miR-142-5p | GCCCCATAAAGTAGAAAGCACTA | |
| bta-miR-27a-3p | TTCACAGTGGCTAAGTTCCG | |
| bta-miR-200b | GCTAATACTGCCTGGTAATGATGA | |
| bta-miR-423-3p | AGCTCGGTCTGAGGCCCCTCA | |
| bta-miR-26a | TTCAAGTAATCCAGGATAGGCT | |
| bta-miR-193a-5p | GTCTTTGCGGGCGAGATGA | |
| bta-miR-186 | CAAAGAATTCTCCTTTTGGGCT | |
| bta-miR-339a | TCCCTGTCCTCCAGGAGCTC | |
| bta-miR-320a | AAAAGCTGGGTTGAGAGGGC | |
| bta-miR-486 | TCCTGTACTGAGCTGCCCC | |
| bta-miR-1 | GCGCTGGAATGTAAAGAAGTATGTAT | |
| bta-miR-11987 | GCAGGAAACTCTGGTGGAGGT | |
| bta-miR-126-5p | CATTATTACTTTTGGTACGCG | |
| bta-miR-2387 | TGGAAGGCCTGGCTTTGCA | |
| *β-actin* | GCACCACACCTTCTACAA | TACGACCAGAGGCATACA |
| *SLC27A1* | GGCGTGGGTCAGTGTCTCAT | CTTCAGCAGGTAGCGGCAGAT |
| *APOA5* | CGCCTTCACTCACGCCATTG | CTCTGTCGGCTTGGAGGAACT |
| *HSL* | CGCAGCAATGAACAGAGACA | TGGTTGGTCCTTGAGCAGAT |
| *FASN* | CACGAACAACAGCCTCTT | GCCTCCAGCACTCTACTA |
| *SCD* | CAACTACCACCACACCTT | TCATAAGCCAGACCGATG |
| *PPARG* | AAAGCGTCAGGGTTCCACTAT | ATCTCCGCTAACAGCTTCTCC |
| *ELOVL6* | ACTGTGCTTCTGTACTCCTGGT | CGTGATGAACATGGCGAACTTC |
| *GPAM* | GCATTGACCCTTGGCACGAT | CGGCTCATTAGGCTTTCTTTCC |
| *CPT1B* | TGTCTGTGTCCGCCTTCTGT | TCATCATCCAGCAAGTGTTCCA |
| *ATGL* | GCTGCACAAAGGCTGCTTCTA | ATGCCAGTCTCGTTTCGTTTGTA |
| *EHHADH* | GCCTGGCACTGATCCGTCTA | TCGGCACCTGCACAGAAGAT |
| *DGAT1* | GGACACAGACAAGGACGGAGA | ATCAGCATCACCACACACCAAT |
| *SREBP1* | CGACACCACCAGCATCAACCACG | GCAGCCCATTCCATCAGCCAGACC |

**Table S2** The sequence of shRNA

| **Pimers** | **Sequences (5´→3´)** |
| --- | --- |
| sh*APOA5*-695-F | GATCCCCAGCAAGCTCACGCTCAACTTCCTGTCAGATTGAGCGTGAGCTTGCTGGTTTTTG |
| sh*APOA5*-695-R | AATTCAAAAACCAGCAAGCTCACGCTCAATCTGACAGGAAGTTGAGCGTGAGCTTGCTGGG |
| sh*APOA5*-792-F | GATCCCGGTGACGATGCGGAGGAACTTCCTGTCAGATTCCTCCGCATCGTCACCGTTTTTG |
| sh*APOA5*-792-R | AATTCAAAAACGGTGACGATGCGGAGGAATCTGACAGGAAGTTCCTCCGCATCGTCACCGG |
| sh*APOA5*-983-F | GATCCCGGAGTTCCTCCAAGCCGACTTCCTGTCAGATCGGCTTGGAGGAACTCCGTTTTTG |
| sh*APOA5*-983-R | AATTCAAAAACGGAGTTCCTCCAAGCCGATCTGACAGGAAGTCGGCTTGGAGGAACTCCGG |
| sh*SLC27A1*-842-F | GATCCCAGCAGATGTGCTCTACGACTTCCTGTCAGATCGTAGAGCACATCTGCTGTTTTTG |
| sh*SLC27A1*-842-R | AATTCAAAAACAGCAGATGTGCTCTACGATCTGACAGGAAGTCGTAGAGCACATCTGCTGG |
| sh*SLC27A1*-727-F | GATCCCGACTCTTCTACATCTACACTTCCTGTCAGTTGTAGATGTAGAAGAGTCGTTTTTG |
| sh*SLC27A1*-727-R | AATTCAAAAACGACTCTTCTACATCTACATCTGACAGGAAGTTGTAGATGTAGAAGAGTCGG |
| sh*SLC27A1*-600-F | GATCCGGTCAAGTTCTGTTCTGGACTTCCTGTCAGATCCAGAACAGAACTTGACCTTTTTG |
| sh*SLC27A1*-600-R | AATTCAAAAAGGTCAAGTTCTGTTCTGGATCTGACAGGAAGTCCAGAACAGAACTTGACCG |

**Table S3** Dual-luciferase reporter vector DNA sequences of candidate target genes

| **Primers** | **Forward sequences (5´→3´)** | **Reverse sequences (5´→3´)** |
| --- | --- | --- |
| APOA5-WT-F | CTAGCCTCCCCATCTCTGGCCCCUCCCTCCTGACTTCC | |
| APOA5-WT-R | CGGGAAGTCAGGAGGGGGGGCCAGAGATGGGGAGG | |
| APOA5-mut-F | CTAGCCTCCCCATCTCTGGCTCATGCCCTCCTGACTTCC | |
| APOA5-mut-R | CGGGAAGTCAGGAGGGCATGAGCCAGAGATGGGGAGG | |
| MECR-WT-F | CTAGCGGCCTGCCTCCGCCTCCTTCTCTGCCTCTGAGCCTTCCCGGGTC | |
| MECR-WT-R | CGGACCCGGGAAGGCTCAGAGGCAGAGAAGGAGGCGGAGGCAGGCCG | |
| MECR-mut-F | CTAGCGGCCTGCCTCCGCGTCGCTATCTCAGCTGCAGCCTTCCCGGGTC | |
| MECR-mut-R | CGGCCGGACGGAGGCGCAGCGATAGAGTCGACGTCGGAAGGGCCCAG | |
| AGPAT4-WT-F | CTAGGAGCCCAGGTGCCCCTCTGTACAGGCTTGG | |
| AGPAT4-WT-R | CGCCAAGCCTGTACAGAGGGGCACCTGGGCTC | |
| AGPAT4-mut-F | CTAGGAGCCCAGGTGCTCATCAGTACAGGCTTGG | |
| AGPAT4-mut-R | CGCCAAGCCTGTACTGATGAGCACCTGGGCTC | |
| SLC27A1-WT-F | CTAGAAGTCCAAATGAGATTTCTCAGGGTCGGAGCATGGCT | |
| SLC27A1-WT-R | CGAGCCATGCTCCGACCCTGAGAAATCTCATTTGGACTT | |
| SLC27A1-mut-F | CTAGAAGTCCAAATGAGATTTACGCGAGTCGGAGCATGGCT | |
| SLC27A1-mut-R | CGGTGCTCCTGAGCTGAGTGAGCAGTGATCCTAC | |
| SLC27A4-WT-F | CTAGGGTGTGGGGCACTCAGGGGCAAATAAAC | |
| SLC27A4-WT-R | CGGTTTATTTGCCCCTGAGTGCCCCACACC | |
| SLC27A4-mut-F | CTAGGGTGTGGCTGTCATGTGCGCAAATAAAC | |
| SLC27A4-mut-R | CGGTTTATTTGCGCACATGACAGCCACACC | |
| AGPAT2-WT-F | CTAGCCAGCTGGGCCTCAGGGAGCCACAAAGC | |
| AGPAT2-WT-R | CGGCTTTGTGGCTCCCTGAGGCCCAGCTGG | |
| AGPAT2-mut-F | CTAGCCAGCTGTCAGTCCTGATGCCACAAAGC | |
| AGPAT2-mut-R | CGGCTTTGTGGCATCAGGACTGACAGCTGG | |

**Table S4** Summary of DHI data for candidate Holstein cows

| **Milk quality traits** | **HMF_group**  **(*n* = 10)** | **LMF_group**  **(*n* = 10)** |
| --- | --- | --- |
| Milk yield, kg/d | 44.63±6.91 | 32.17±2.41 |
| Milk fat percentage, % | 5.71±0.027 | 2.07±0.35 |
| 4% FCM, kg/d | 56.06±13.81 | 22.83±2.28 |
| Milk protein percentage, % | 3.47±0.086 | 3.21±0.18 |
| Milk fat/milk protein | 1.65±0.02 | 0.64±0.11 |
| SCC ,10^4^/mL | 9.20±4.62 | 3.20±2.14 |
| SCS | 2.0±0.44 | 1.30±1.27 |
| MUN, mg/dL | 18.71±13.75 | 13.82±1.41 |

FCM: Fat-corrected milk; SCC: Somatic cell count; SCS: Somatic cell scores; MUN: Milk urea nitrogen

**Table S5** Detailed DHI data for candidate Holstein cows

| **Group** | **Individual** | **Milk yield, kg/d** | **MFP, %** | **4% FCM, kg/d** | **MPP, %** | **Milk fat/milk protein** | **SCC, 10^4^/mL** | **SCS** | **MUN content, mg/dL** |
| --- | --- | --- | --- | --- | --- | --- | --- | --- | --- |
| **HMF group**  **(*n* = 10)** | 170259 | 46.5 | 5.69 | 58.29 | 4.14 | 1.37 | 8 | 2 | 22.5 |
|  | 170549 | 48.1 | 5.83 | 61.30 | 3.25 | 1.79 | 7 | 2 | 24.3 |
|  | 171239 | 42.7 | 5.5 | 52.31 | 3.56 | 1.54 | 10 | 3 | 22.6 |
|  | 180013 | 42.3 | 5.59 | 52.39 | 3.43 | 1.63 | 12 | 2 | 17.7 |
|  | 180073 | 47.9 | 5.93 | 61.77 | 3.12 | 1.9 | 9 | 2 | 16 |
|  | 180355 | 43.2 | 5.65 | 53.89 | 3.35 | 1.69 | 10 | 1 | 12.7 |
|  | 180383 | 46.5 | 5.61 | 57.73 | 3.28 | 1.71 | 11 | 2 | 15.7 |
|  | 180496 | 45.8 | 5.53 | 56.31 | 3.3 | 1.68 | 7 | 1 | 20.8 |
|  | 180571 | 40.6 | 5.74 | 51.20 | 3.65 | 1.57 | 6 | 2 | 16.5 |
|  | 190068 | 42.7 | 5.98 | 55.38 | 3.65 | 1.64 | 12 | 3 | 18.3 |
| **LMF group**  **(*n* = 10)** | 190001 | 29.3 | 2.31 | 21.87 | 3.45 | 0.67 | 2 | 0 | 14 |
|  | 190012 | 31.4 | 1.52 | 19.72 | 3.01 | 0.5 | 0 | 3 | 11.7 |
|  | 190042 | 31.4 | 2.42 | 23.96 | 3.14 | 0.77 | 1 | 0 | 15.5 |
|  | 190159 | 35.1 | 2.18 | 25.52 | 3.24 | 0.67 | 6 | 3 | 12.9 |
|  | 190160 | 29.9 | 2.41 | 22.77 | 3.03 | 0.8 | 2 | 1 | 12.3 |
|  | 190182 | 28.5 | 2.24 | 20.98 | 3.2 | 0.7 | 5 | 0 | 12.5 |
|  | 190194 | 33.5 | 1.61 | 21.49 | 3.47 | 0.46 | 3 | 0 | 14.6 |
|  | 190220 | 34.4 | 2.1 | 24.60 | 3.4 | 0.62 | 4 | 2 | 16 |
|  | 190355 | 32.2 | 1.53 | 20.27 | 2.92 | 0.52 | 2 | 3 | 13.4 |
|  | 190415 | 36 | 2.35 | 27.09 | 3.22 | 0.73 | 7 | 1 | 15.3 |

**Table S6** The table of sample data quality control

| **#Sample** | **Total_Reads** | **Total_Bases** | **A%** | **T%** | **C%** | **G%** | **Q20%** | **Q30%** | **GC%** |
| --- | --- | --- | --- | --- | --- | --- | --- | --- | --- |
| HMF  _EXO | 20,003,230 | 1,020,164,730 | 21.98 | 25.18 | 25.18 | 33.71 | 99.42 | 96.53 | 58.89 |
| LMF  _EXO | 18,982,930 | 968,129,430 | 22.34 | 22.34 | 26.35 | 29.58 | 96.53 | 96.53 | 55.93 |

**Figures**


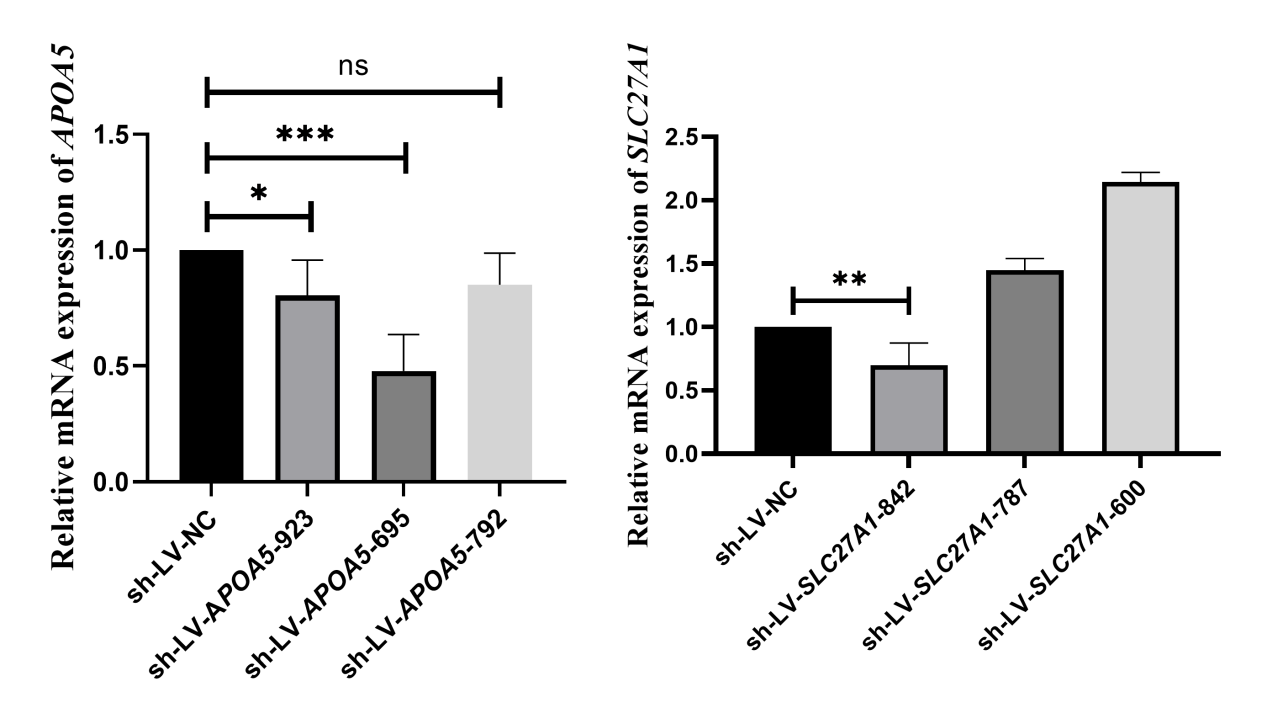


**Fig. S1** Validation of interference efficiency of LV-sh*APOA5*/sh*SLC27A1* vector.

^***^*P* < 0.001, ^**^*P* < 0.01, ^**^*P* < 0.05, ns: not significant

**
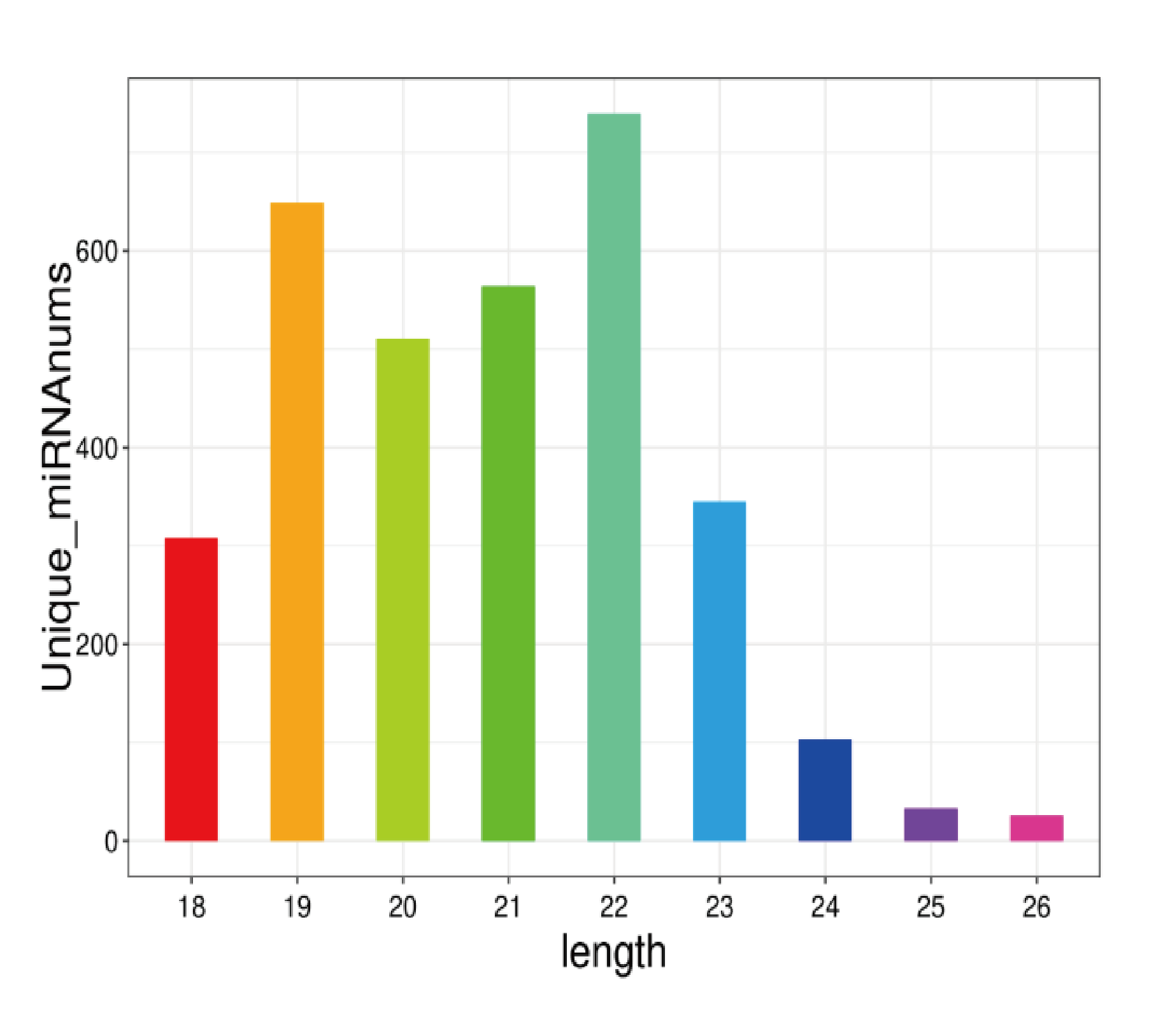
**

**Fig. S2** The length distribution of high-quality clean data

**
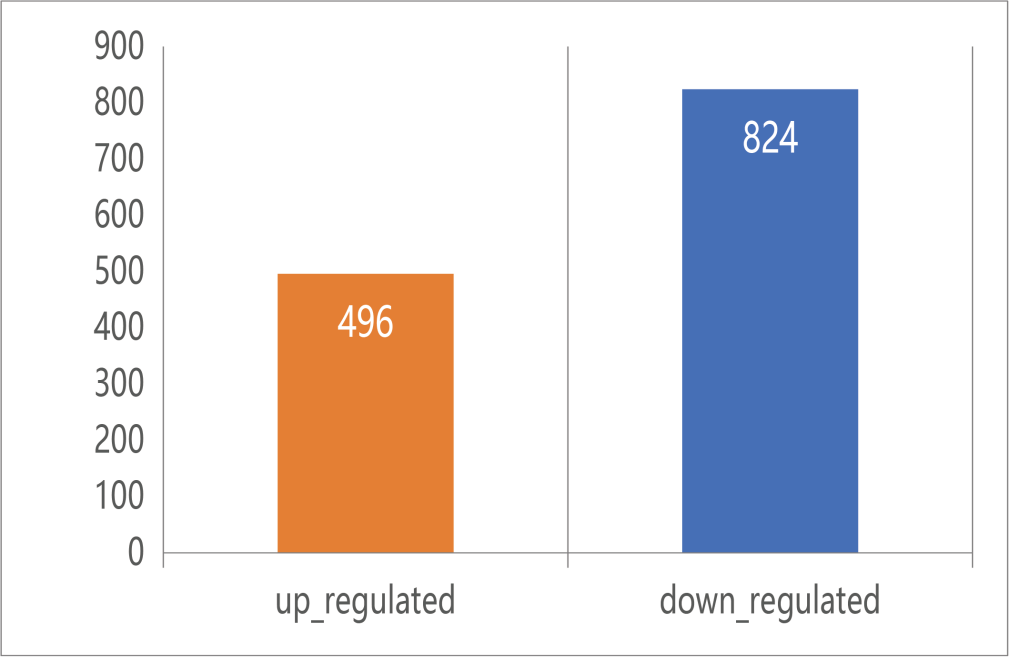
**

**Fig. S3** The number of differentially expressed miRNAs between the two groups
